# Do studies of interventions to improve laypeople’s critical thinking about health choices assess potential harms? A systematic review

**DOI:** 10.1136/bmjopen-2025-108268

**Published:** 2026-04-24

**Authors:** Matt Oxman, Leila Cusack, Francisca Verdugo-Paiva, Camila Ávila, Javiera Peña, Francisco Novillo, Andrew D Oxman, Atle Fretheim, Monica Melby-Lervåg, Lena Nordheim

**Affiliations:** 1 Centre for Epidemic Interventions Research, Norwegian Institute of Public Health, Oslo, Norway; 2 Oslo Metropolitan University Faculty of Health Sciences, Oslo, Norway; 3 Institute for Evidence-Based Healthcare, Bond University, Robina, Queensland, Australia; 4 Epistemonikos Foundation, Santiago, Chile; 5 Facultad de Odontología, Universidad Andrés Bello, Santiago, Chile; 6 Universidad San Sebastián Facultad de Odontología y Ciencias de la Rehabilitación, Santiago, Chile; 7 School of Dentistry, Pontificia Universidad Católica de Chile Facultad de Medicina, Santiago, Chile; 8 Centre for Research on Equality in Education, University of Oslo Department of Special Education, Oslo, Norway; 9 Department of Health and Functioning, Western Norway University of Applied Sciences Faculty of Health and Social Sciences, Bergen, Norway

**Keywords:** PUBLIC HEALTH, Health Education, Health Literacy, Systematic Review

## Abstract

**Abstract:**

**Objectives:**

To make informed health choices, and avoid waste and unnecessary suffering, people need critical thinking skills. However, like health interventions, educational interventions can have adverse effects. In this systematic review, the objective was to assess the extent to which researchers have included potential adverse effects in studies of interventions intended to improve the critical thinking of laypeople about health choices.

**Design:**

This study was a systematic review, in which we updated the search for an earlier systematic review of intended effects of relevant interventions. The earlier review did not address potential adverse effects. We did not update the analysis of intended effects.

**Data sources:**

We searched Cochrane Central Register of Controlled Trials (CENTRAL), Cumulative Index to Nursing and Allied Health Literature (CINAHL), Embase, Epistemonikos, Medical Literature Analysis and Retrieval System Online (MEDLINE), Education Resources Information Center (ERIC) and Web of Science up to March 2025. In addition to studies from the original review and updated search, we included any additional studies included in a similar, even earlier review. Our unit of analysis was study report (eg, journal article).

**Eligibility criteria:**

We included all studies from the original review. We applied the same inclusion criteria to the results of the updated search: the study included a comparison, the population was laypeople and the intervention was intended to improve understanding of ≥1 key concept for informed health choices.

**Data extraction and synthesis:**

We extracted data about study design (randomised trial or other), participants (children, adolescents or adults), study setting (countries), main intervention (resources delivered to participants) and comparator (usual/no intervention or other). For the analysis, we extracted verbatim text describing any assessment of a potential adverse effect of the intervention. We conducted a narrative synthesis of the extracted data.

**Results:**

We included 35 reports of quantitative studies (including multi-method and mixed-methods studies). Most often, the study was a randomised trial, the setting was a high-income country, the population included adults (including university students) and the intervention was school-based (including university). In one of the 35 reports, authors described assessing a potential adverse effect.

**Conclusion:**

To our knowledge, this is the first systematic review assessing the extent to which researchers have assessed adverse effects of any category of educational interventions. Our review shows that researchers generally have not assessed potential adverse effects of interventions to improve critical thinking about health choices. Researchers should pay more attention to such effects, while policymakers and educators making decisions about implementing relevant interventions should consider the lack of evidence. The findings of this study suggest a need for research that facilitates assessing potential adverse effects of interventions to improve critical thinking about health choices.

**STRENGTHS AND LIMITATIONS OF THIS STUDY**

- We updated an extensive search for an earlier systematic review.
- In addition to quantitative studies, we assessed associated qualitative studies and protocols for both the quantitative and qualitative studies.
- This was an ad hoc study, but the original review was pre-registered with a protocol.
- We did not assess adverse effects, but this would not have been useful due to the lack of evidence.

## Introduction

 Every day, people make health choices, defined here as decisions about whether to do something for the health of an individual or group, that is, choices about health interventions. A health intervention is any action intended to improve or maintain the health of individuals or groups, including, but not limited to, ‘modern’ and ‘alternative’ medicine, lifestyle interventions and health policies.^1 2^ Health interventions can have beneficial, but also adverse effects.^3^ An adverse effect (harm) of an intervention is an increase in adverse (undesirable) outcomes, or a decrease in beneficial (desirable) outcomes, caused by the intervention. For example, in a systematic review of adverse effects of herbal medicines, researchers found the most common adverse effects were increases in digestive symptoms such as indigestion, nausea and diarrhoea.^4^

The harms of a health intervention sometimes outweigh the benefits.^5^ To make informed health choices and choose interventions with benefits that outweigh any harms, people need critical thinking skills.^6 7^ There are various definitions of critical thinking.^8^ For example, Ennis has defined critical thinking as ‘reasonable reflective thinking focused on deciding what to believe or do’.^9^ In terms of health choices, ADO *et al* have iteratively identified and formulated concepts involved in critical thinking and organised them in a framework called Key concepts for informed health choices (also known as the Informed Health Choices key concepts).^6 7^ The ability to apply the concepts is part of critical health literacy.^10^

In the last version of the framework, the concepts are organised in three main groups (table 1).^7^ Within the groups, there is a total of 10 overarching ‘high-level’ concepts, and within the high-level concepts, there is a total of 49 elemental concepts, such as the difference between correlation and causation.^3 7^ Many of the key concepts for informed health choices are applicable to other fields.^11^

**Table 1:** Key concepts for informed health choices: groups and high-level concepts^7^

Group	1. Claims	2. Comparisons	3. Choices
Description of group	Claims about effects that are not supported by evidence from fair comparisons are not necessarily wrong, but there is an insufficient basis for believing them	Studies should make fair comparisons, designed to minimise the risk of systematic errors (biases) and random errors (the play of chance)	What to do depends on judgements about a problem, the relevance of the available evidence and the balance of expected benefits, harms and costs.
High-level concepts	1.1 Assumptions that treatments are safe or effective can be misleading 1.2 Seemingly logical assumptions about research can be misleading 1.3 Seemingly logical assumptions about treatments can be misleading 1.4 Trust based on the source of a claim alone can be misleading	2.1 Comparisons of treatments should be fair 2.2 Reviews of the effects of treatments should be fair 2.3 Descriptions of effects should clearly reflect the size of the effects 2.4 Descriptions of effects should reflect the risk of being misled by the play of chance	3.1 Evidence should be relevant 3.2 Expected advantages should outweigh expected disadvantages

Researchers and others have developed and evaluated a variety of educational interventions to improve the ability of laypeople to apply key concepts for informed health choices.^12^,^14^ In a systematic review, LC *et al*^12^ assessed the intended effects of such interventions in laypeople (non-health professionals) but did not address potential adverse effects of the interventions. However, like health interventions, educational interventions can have adverse effects.^1115^,^18^

Assessing intended effects of interventions can reveal paradoxical effects.^19 20^ For example, in a randomised trial of a whole-school improvement programme called Achievement for All, researchers found a paradoxical effect on progress in reading (progress was slower in the intervention arm).^21^ Besides paradoxical effects, educational interventions might have other adverse effects.^11 15 17 18 22 23^ For example, in a qualitative study linked to a trial of a secondary school intervention to improve critical thinking about health choices, MO *et al* found that a small number of participants suggested the intervention might cause inequities or exacerbate inequities, depending on factors such as access to reliable health information.^24^

In this systematic review, the research question was: do studies of interventions to improve critical thinking about health choices assess potential adverse effects? We included all the studies in the earlier review by LC *et al*^12^ and updated the search and study selection. We did not update the analysis of intended effects.

## Methods

Online supplemental file 1 is the Preferred Reporting Items for Systematic Reviews and Meta-Analyses (PRISMA) checklist for this review. Online supplemental file 2 is the PRISMA checklist for abstracts. The review by LC *et al*^12^ was pre-registered with a protocol in the International Prospective Register of Systematic Reviews (PROSPERO; no. CRD42016033103). There was no protocol specifically for this study.

### Eligibility criteria

We used the same eligibility criteria as in the original review by LC *et al*.^12^ The inclusion criteria are summarised in table 2. Like in the original review, we only included primary studies. We did not add eligibility criteria about adverse effects because it seemed unlikely that there would be eligible studies of potential adverse effects only and not intended effects.

**Table 2:** Inclusion criteria for the review

**Category**	Criteria
Study design	Any primary, quantitative study design that includes a comparison (randomised trials, non-randomised trials with concurrent controls, controlled before and after studies, controlled studies with only post-test measures and interrupted time series studies)
Population	All laypeople, including children and adolescents
Intervention	Any intervention intended to help the participants (learners) understand ≥1 key concepts for informed health choices
Comparator	Any intervention or no intervention
Outcome	Understanding of ≥1 key concept for informed health choices
Publication	Any type and language

Laypeople were defined as any population except health professionals (eg, nurses) or university students in health professional programmes (eg, a bachelor’s programme in nursing). Studies with a mix of eligible and ineligible populations (eg, a mix of patients and nurses or nursing students) were eligible if the results for laypeople were reported or available separately. Students taking a university course about health, but not enrolled in a health professional programme, were eligible.

Studies were ineligible if the examples and scenarios used in the intervention were not about health. Relevance of the intervention could be indicated by a general topic representing >1 concept (eg, scientific reasoning or critical health literacy) or a single concept (eg, randomisation or blinding). Studies of interventions to improve informed consent about participating in a specific clinical trial were ineligible.

### Search strategy

We twice updated the search for the original review. In the first update, we searched Cochrane Central Register of Controlled Trials (CENTRAL), Cumulative Index to Nursing and Allied Health Literature (CINAHL), Embase and Medical Literature Analysis and Retrieval System Online (MEDLINE), up to December 2022, Education Resources Information Center (ERIC) up to March 2023 and Web of Science up to May 2023. In addition, we searched the Epistemonikos database up to December 2022. Epistemonikos is a comprehensive database of systematic reviews, maintained through regular automated searches across multiple sources, including the Cochrane Database of Systematic Reviews, PubMed/MEDLINE, Embase, CINAHL, PsycINFO, Latin American and Caribbean Health Sciences Literature (LILACS), Database of Abstracts of Reviews of Effects (DARE), the Campbell Library, the Joanna Briggs Institute (JBI) Database of Systematic Reviews and Implementation Reports and the Evidence for Policy and Practice Information (EPPI)-Centre Evidence Library. In the second update, we searched the same sources as in the first update, this time up to March 2025.

We adapted the Boolean strategy to the syntax of each source to account for differences in indexing terms and search functionalities. We did not add terms for adverse effects (eg, ‘adverse effect’ or ‘harm’) to the search strings, because the original search was not limited to studies of intended or beneficial effects. Adding terms for potential adverse effects would only reduce the number of search results. Moreover, as noted in the section on eligibility criteria, it seemed unlikely that there would be eligible studies of potential adverse effects only and not intended effects. Online supplemental file 3 shows the detailed search strings for the second update.

We did not search for grey literature, qualitative studies or protocols (see Selection process).

### Selection process

We uploaded records of potentially eligible studies to the Sustainable Knowledge (SK) Platform (https://www.skplatform.org/) (Epistemonikos, Santiago, Chile), a suite of interconnected tools designed to support evidence synthesis processes. We deduplicated records using the SK screening tool, which uses an algorithm that compares unique identifiers (eg, database ID, DOI, trial registry ID) and citation metadata (eg, author names, journal title, publication year, volume, issue, page numbers, article title and abstract).

Following deduplication, we provided the inclusion and exclusion criteria for the review within the SK platform. We aligned components of the review question with artificial intelligence classifiers embedded in the SK platform. We then applied classifiers to exclude records deemed irrelevant based on titles and abstracts. Two reviewers independently screened the remaining records at the title and abstract level. A third reviewer resolved disagreements.

We retrieved full texts for all studies that appeared to meet the inclusion criteria or required further evaluation. Two reviewers independently assessed each full text for eligibility, with a third reviewer resolving disagreements. Reasons for exclusion at the full-text stage are documented in online supplemental file 4.

In addition to the study reports from the original review by LC *et al* and from the updated search, we included any additional study reports included in a similar, earlier review by LN *et al* of the effects of school-based interventions to enhance the abilities of adolescents to critically appraise health claims.^13^ This was an opportunistic decision, to identify eligible studies we had missed or—if additional studies included by LN *et al* did not meet our eligibility criteria—to assess the extent to which potential adverse effects are included in a wider, but still similar group of studies. LN *et al* based their eligibility criterion for interventions on learning aims for science education in general, identified by Ryder,^25^ which are broader than the key concepts for informed health choices. For each study included by LN *et al* that was excluded by LC *et al* in their review, we confirmed if the study report had appeared in the search by LC *et al*, and why it did not appear in their search or why they had excluded it.

In our review, two investigators checked all included reports of quantitative studies for references to separate reports of associated qualitative studies—for example, “We report a process evaluation in a separate paper”.^26^ If there was such a reference, MO retrieved the referenced report and we included it. By associated qualitative study, we mean a process evaluation or other qualitative study conducted alongside or after the quantitative study, to complement the quantitative study.^27 28^

Two reviewers checked the included reports of both quantitative and qualitative studies for references to protocols. If there was such a reference, and it included the location of a publicly available copy of the protocol, MO retrieved the protocol.

### Data extraction

MO developed an extraction spreadsheet for study characteristics.^29^ We extracted data about study design (randomised trial or other), participants (children, adolescents or adults), study setting (countries), main intervention (resources delivered to participants) and comparator (usual/no intervention or other).

We categorised participants as children if their age was <13 years, adolescents if their age was 13–18 years and adults if their age was >18 years. If the report did not include the age range of participants, we categorised participants as children if they were in primary (elementary) school, adolescents if they were in secondary (middle or high) school and adults if they were in university. If the report did not include the age range of participants, and the intervention was not school-based, we made a judgement about participant category based on terminology used in the report (eg,‘children’) or on other reported information (eg, mean or median age of participants or their occupation).

The terms we used to describe resources delivered to participants as part of main interventions (eg, ‘curriculum’) were based on the terminology used in the respective reports, not any categorisation on our end.

MO developed a separate extraction spreadsheet for data about potential adverse effects for the analysis, with one sheet each for reports of quantitative and qualitative studies, and one for protocols.^30^ The data were verbatim text in the reports mentioning potential adverse effects of the interventions. We based judgements about what to extract on the use of relevant terminology (eg, ‘adverse effect’, ‘harm’, ‘undesirable effect’, ‘side effect’ and ‘negative effect’) and our definition of an adverse effect of an intervention (an increase in adverse outcomes or decrease in beneficial outcomes caused by the intervention), but not the specific use of ‘adverse effect’ or other terms. The effect could be an increase in any adverse outcome or decrease in any beneficial outcome. We took the descriptions of effects by authors as adverse at face value.

From reports of quantitative studies, including multi-method or mixed-methods studies (eligible studies in which the researchers used both quantitative and qualitative methods), we extracted:

- Descriptions of methods used to assess potential adverse effects specifically, and results from assessing such effects.
- Descriptions of the use of qualitative methods of data collection and analysis (in addition to the quantitative methods).
- References to separate reports of process evaluations or other associated qualitative studies.
- References to protocols for the quantitative studies and if a protocol was publicly available, where (eg, trial registry).

By assessing a potential adverse effect specifically, we mean measuring and comparing outcomes in an intervention and control arm specifically to assess a potential adverse effect. In other words, this excludes assessing intended effects, even if assessing the intended effect reveals a paradoxical adverse effect.

From reports of multi-method and mixed methods studies and associated qualitative studies, we extracted relevant qualitative findings, as well as references to protocols for the qualitative studies and if a protocol was publicly available, where. Like with reports of quantitative studies, we made judgments about what findings to extract based on the use of relevant terminology, and our definition of an adverse effect, but not the specific use of any term. Qualitative findings were relevant if the research questions or aims stated in the report explicitly included potential adverse effects, and the findings were explicitly about potential adverse effects. In other words, we did not reinterpret findings. However, when extracting data, we erred on the side of extracting ‘too much’ (ie, extracting findings that we would probably agree not to include in the analysis).

From protocols, we extracted any mention of potential adverse effects. Again, we based data extraction on relevant terminology and our definition of adverse effects (increases in adverse outcomes, or decreases in beneficial outcomes), not the specific use of certain terms. From both study reports and protocols, we extracted additional data that were related to our aims and analysis, but not used in the analysis—for example, information about risk assessments or safety monitoring—to ensure we agreed those data should not be included in the analysis, and to provide additional context.

At every stage, two reviewers independently extracted data, then harmonised what they each had extracted, resolving any inconsistencies. To extract data from reports or protocols not in English, we used Google Translate (www.translate.google.com), as well as recruited a researcher fluent in the other language to independently extract and translate any relevant data.

### Analysis

In our primary analysis, we assessed the reports of quantitative studies. In a secondary analysis, we assessed any reports of associated qualitative studies. In another secondary analysis, we assessed any protocols for the quantitative studies and any protocols for the qualitative studies. We did not conduct a meta-analysis or qualitative evidence synthesis, given the aim of our review was to assess the extent to which potential adverse effects are included in studies of relevant interventions, not to assess or explore experiences of such effects. Rather, we analysed the data using descriptive statistics and conducted a narrative synthesis. The unit of analysis was study report or protocol. By study report, we mean a written report of a completed research study (eg, journal article, thesis, book chapter and poster). If the report of a quantitative study included a description of the use of qualitative methods of data collection and analysis (in addition to quantitative methods), we considered the study multi-method or mixed methods and counted the report as both a report of a quantitative study and a report of a qualitative study. Some of the process evaluations and other associated qualitative studies were in fact multi-method or mixed methods, but did not include a comparison between interventions (table 2). For the purposes of this review, we considered those studies purely qualitative, meaning we did not include the reports of those studies in the analysis of quantitative study reports.

## Results

### Study selection

**Figure 1:**
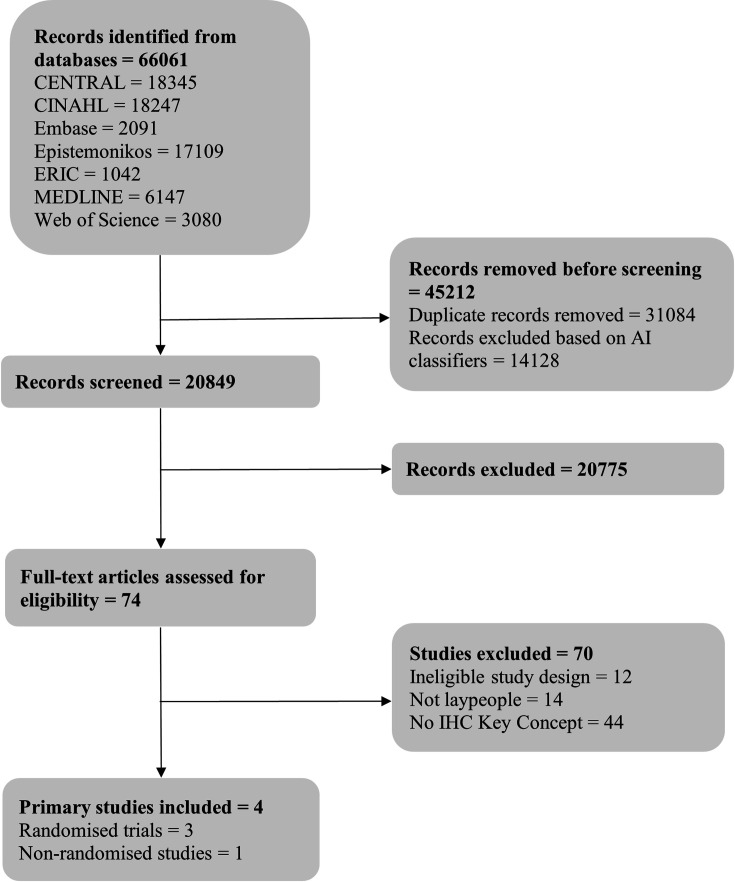
Preferred Reporting Items for Systematic Reviews and Meta-Analyses flow diagram for the first search update. CENTRAL, Cochrane Central Register of Controlled Trials; CINAHL, Cumulative Index to Nursing and Allied Health Literature; ERIC, Education Resources Information Center; MEDLINE, Medical Literature Analysis and Retrieval System Online; AI, artificial intelligence; IHC, Informed Health Choices.

**Figure 2:**
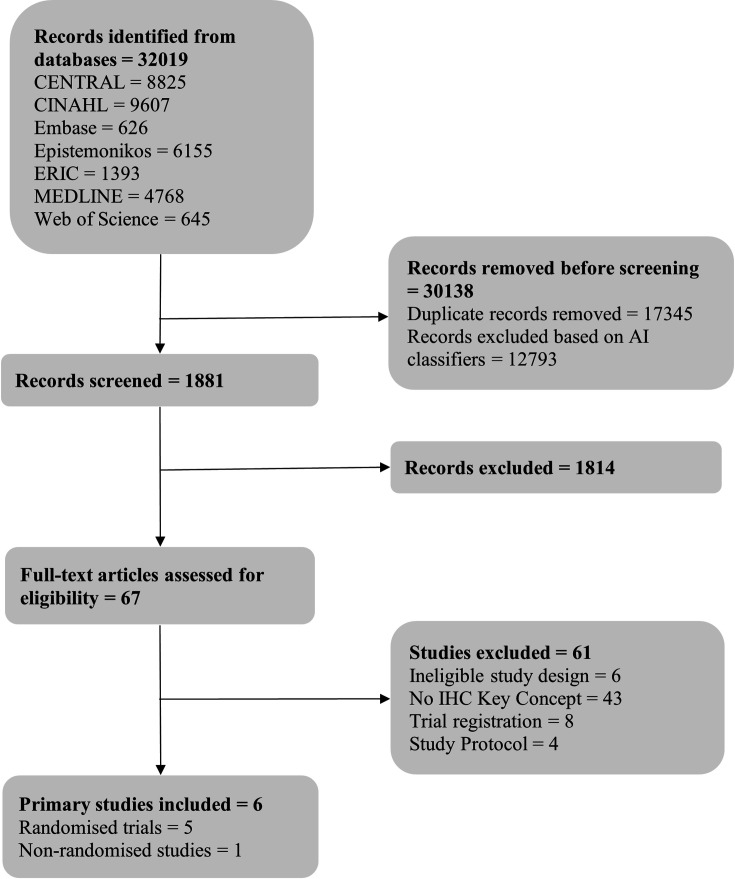
Preferred Reporting Items for Systematic Reviews and Meta-Analyses flow diagram for the second search update. CENTRAL, Cochrane Central Register of Controlled Trials; CINAHL, Cumulative Index to Nursing and Allied Health Literature; ERIC, Education Resources Information Center; MEDLINE, Medical Literature Analysis and Retrieval System Online; AI, artificial intelligence; IHC, Informed Health Choices.

The first update to the search yielded 66 061 references. After removing duplicates and excluding records with a low probability of satisfying the inclusion criteria, based on the automated classifiers, we screened a total of 20 849 records by title and abstract. We retrieved full texts for 74 studies that appeared to meet the inclusion criteria or required further assessment. Based on full-text evaluation, 4 of the 74 study reports met the eligibility criteria: 3 reports of randomised controlled trials^26 31 32^ and 1 report of a non-randomised study.^33^ The second update to the search yielded 32 019 references. After excluding duplicates and records with a low probability of matching the inclusion criteria, we screened 1881 records by title and abstract. We retrieved full texts for 67 studies, of which 6 satisfied the inclusion criteria: 5 reports of randomised trials^34^,^38^ and 1 of a non-randomised study.^39^ The study selection processes for both search updates are presented in separate PRISMA flow diagrams (figure 1 and figure 2).

LN *et al* included seven study reports^1340^,^46^ in their review.^13^ LC *et al* excluded three of those reports^41 43 45^ from their later review.^12^ They excluded one that was a thesis^41^ because the study was also reported in a later journal article that they included instead.^47^ They excluded the other two^43 45^ because the interventions were not intended to improve understanding of any specific key concept for informed health choices.

Altogether, in this review, we included 35 reports of quantitative studies: all 22 included in the original review^12^; 10 additional study reports identified in the updated searches (4 from the first update and 6 from the second update); and 3 additional study reports included in the review by LN *et al*.^13^ Table 3 is an overview of the reports of quantitative studies included in this review. The underlying data include a copy of the data extraction spreadsheet with all data extracted for the analysis.^29^

**Table 3:** Included reports of quantitative studies

Study report	Assessment of potential harms	Multi-method or mixed-methods	Reference to associated qualitative study	Reference to protocol	Location of publicly available protocol
Alderighi *et al*^39^	No	Yes	No	Yes	No
Austvoll-Dahlgren *et al*^55^	Yes	No	No	Yes	Supplementary material
Barnett *et al*^66^	No	No	No	No	Not applicable
Berger *et al*^60^	No	Yes	No	No	Not applicable
Chesire *et al*^35^	No	No	Cheshire *et al*^48^	Yes	General-purpose repository
Ciarocco *et al*^67^	No	No	No	No	Not applicable
Cusack *et al*^34^	No	Yes	No	Yes	Supplementary material
Derry *et al*^40^	No	Yes	Osana *et al*^68^	No	Not applicable
Dunn *et al*^69^	No	Yes	No	Yes	No
Hendricks^47^	No	Yes	No	No	Not applicable
Hill^41^	No	Yes	No	No	Not applicable
Jacque *et al*^33^	No	No	No	No	Not applicable
Kaelin *et al*^42^	No	No	No	Yes	No
Kruse *et al*^70^	No	No	No	No	Not applicable
Kuhn *et al*^43^	No	Yes	No	No	Not applicable
Leshowitz *et al*^44^	No	No	No	No	Not applicable
Leshowitz *et al*^71^	No	No	No	No	Not applicable
Matic-Strametz *et al*^72^	No	No	No	No	Not applicable
Mugisha *et al*^37^	No	No	Mugisha *et al*^49^	Yes	General-purpose repository
Ndebele *et al*^73^	No	No	No	No	Not applicable
Nsangi *et al*^53^	No	No	Nsangi *et al*^50^	Yes	Journal
Nsangi *et al*^26^	No	No	Nsangi *et al*^50^	Yes	Journal
Okabayashi *et al*^38^	No	No	No	Yes	Trial registry
Ouimet *et al*^74^	No	No	No	Yes	No
Powell^45^	No	Yes	No	Yes	No
Rowe *et al*^75^	No	No	No	No	Not applicable
Santesso *et al*^76^	No	No	No	Yes	No
Semakula *et al*^54^	No	No	Semakula *et al*^51^	Yes	Journal
Semakula *et al*^31^	No	No	Semakula *et al*^51^	Yes	Journal
Ssenyonga *et al*^36^	No	No	Ssenyonga *et al*^52^	Yes	General-purpose repository
Steckelberg *et al*^46^	No	Yes	Steckelberg *et al*^59^	No	Not applicable
Tait *et al*^77^	No	No	No	No	Not applicable
Welch *et al*^78^	No	No	No	No	Not applicable
Woloshin *et al*^79^	No	No	No	Yes	Trial registry
Wronowski *et al*^32^	No	No	No	No	Not applicable

In 10 of the 35 reports (29%) of quantitative studies, the authors also reported the use of qualitative data collection and analysis, that is, we considered those studies multi-method or mixed-methods (table 3) and counted those reports as both reports of quantitative studies and reports of qualitative studies. Nine of 35 reports of quantitative studies (26%) referenced 7 separate reports of associated qualitative studies (table 3). Seventeen reports of quantitative studies referenced a protocol for the study (table 3). Eleven included the location of a publicly available copy of the protocol (table 3). Six reports of mixed-methods, multi-method or qualitative studies^3948^,^52^ included references to protocols. One of those six reports^39^ did not include the location of a publicly available copy of the protocol.

MO, AF and ADO are co-authors of seven of the included reports of quantitative studies^2631 35^,^37 53 54^ and five of the separate reports of qualitative studies.^48^,^52^ LC is the co-author of one included report of a quantitative study.^34^

### Study characteristics

Characteristics of the included quantitative studies are presented in table 4 and, with additional detail, in the underlying data.^29^ Of the 35 included reports of quantitative studies, 21 were reports of randomised trials. The studies were in 15 different countries, in total. Most of the reports were of studies in high-income countries, including 16 reports of studies in the USA. In 21 reports, the population included adults (including university students), while in 18 reports, the population included children or adolescents. Most of the interventions were school-based (including university). In 17 of the reports, the comparator was no intervention or the usual intervention, including teaching as usual in the case of school-based interventions. Other interventions were generally standard alternatives, such as a standard course.

**Table 4:** Characteristics of quantitative studies

Study report	Study design	Countries	Participants	Main intervention (delivery of resources)	Comparator
Alderighi *et al*^39^	Other	Italy	Children	Learning resources and lessons	Usual/no intervention
Austvoll-Dahlgren *et al*^55^	Randomised trial	Norway	Adults	Web portal	Usual/no intervention
Barnett *et al*^66^	Randomised trial	UK	Children	Information leaflets (three styles)	Not applicable
Berger *et al*^60^	Other	Germany	Adults	Workshop/training course	Other
Chesire *et al*^35^	Randomised trial	Kenya	Adolescents	Digital resources and training workshop	Usual/no intervention
Ciarocco *et al*^67^	Other	USA	Adults	Course	Other
Cusack *et al*^34^	Randomised trial	Australia	Adolescents	Programme materials	Usual/no intervention
Derry *et al*^40^	Other	USA	Adolescents	Instructional unit	Usual/no intervention
Dunn *et al*^69^	Randomised trial	USA	Adults	Module/slides	Other
Hendricks^47^	Randomised trial	USA	Adolescents	Instructional model	Other
Hill^41^	Randomised trial	USA	Adolescents	Instructional model	Other
Jacque *et al*^33^	Other	USA	Adolescents	Curriculum	Usual/no intervention
Kaelin *et al*^42^	Other	USA	Children, adolescents	Curriculum	Usual/no intervention
Kruse *et al*^70^	Randomised trial	Denmark	Adults	Information materials (three types)	Not applicable
Kuhn *et al*^43^	Other	USA	Adolescents	Unit	Usual/no intervention
Leshowitz *et al*^44^	Other	USA	Adolescents, adults	Instructional programme	Usual/no intervention
Leshowitz *et al*^71^	Other	USA	Adults	Instructional programme	Usual/no intervention
Matic-Strametz *et al*^72^	Other	Germany	Adults	Course	Usual/no intervention
Mugisha *et al*^37^	Randomised trial	Rwanda	Adolescents	Digital resources and training workshop	Usual/no intervention
Ndebele *et al*^73^	Randomised trial	Malawi	Adults	Presentation	Other
Nsangi *et al*^53^	Randomised trial	Uganda	Children, adults	Workshop, teaching materials	Usual/no intervention
Nsangi *et al*^26^	Randomised trial	Uganda	Children, adolescents, adults	Workshop, teaching materials	Usual/no intervention
Okabayashi *et al*^38^	Randomised trial	Japan	Adults	E-learning materials	Other
Ouimet *et al*^74^	Other	Canada	Adults	Course	Other
Powell *et al*^45^	Other	USA	Adolescents	Curriculum	Other
Rowe *et al*^75^	Other	USA	Adults	Course	Other
Santesso *et al*^76^	Randomised trial	Canada, Norway, Spain, Argentina, Italy	Adults	Written summary	Other
Semakula *et al*^54^	Randomised trial	Uganda	Adults	Podcast	Other
Semakula *et al*^31^	Randomised trial	Uganda	Adults	Podcast	Other
Ssenyonga *et al*^36^	Randomised trial	Uganda	Adolescents	Digital resources and training workshop	Usual/no intervention
Steckelberg *et al*^46^	Other	Germany	Adolescents	Curriculum	Usual/no intervention
Tait *et al*^77^	Randomised trial	USA	Children, adolescents, adults	Interactive multimedia programme	Other
Welch *et al*^78^	Randomised trial	USA	Adults	Web-based modules	Usual/no intervention
Woloshin *et al*^79^	Randomised trial	USA	Adults	Booklet	Other
Wronowski *et al*^32^	Randomised trial	USA	Adults	Computer game	Other

### Primary analysis

One of the 35 reports of quantitative studies (3%) included the assessment of a potential adverse effect^55^ (table 3). The verbatim methods and results of that assessment are presented in table 5 for illustrative purposes. The intervention was delivering a web portal.^55 56^ The potential adverse effect was an increase in unnecessary pressure to participate in shared decision-making in clinical practice,^57^ assessed using part of a questionnaire based on the Theory of Planned Behaviour model,^58^ specifically the part representing the domain subjective norm (personal perception of social pressure or expectations and motivation to comply).^55^ Presumably, the authors meant that an increase in unnecessary pressure would be captured by the overall score for subjective norm being higher in the intervention arm than the control arm, although they do not specify as much. The researchers did not find such an effect: the mean difference in subjective norm between the intervention and control arms was −0.2 with a 95% CI ranging from −0.8 to 0.4 (p=0.49).^55^

**Table 5:** Verbatim methods and results describing quantitative assessments of potential adverse effects

Study report	Resource	Methods	Results
Austvoll-Dahlgren *et al*^55^	Web portal	A tool to encourage participation could create potentially unnecessary pressure on users; this domain was captured using the Theory of Planned Behaviour questionnaire (subjective norm); other adverse effects were deemed unlikely	The mean differences for the overall subjective norm (20.2; p=0.49) were not statistically significant

### Secondary analyses

In 1 of the 10 reports of multi-method and mixed-methods studies (10%), and 5 of the 7 separate reports of associated qualitative studies (71%), the authors described exploring experiences of potential adverse effects, using qualitative methods. In total, in 6 of the 17 multi-method, mixed-methods or qualitative studies (35%), investigators explored potential adverse effects of the intervention, according to the reports. Table 6 is an overview of the relevant qualitative findings from those six studies. One of the six was reported in an unpublished poster,^59^ a copy of which the first author shared with us on request. In table 6, we have used bold font to highlight what we consider the outcomes of the potential adverse effects of the intervention. In our opinion, in the study by Berger *et al*,^60^ findings about such effects (eg, an increase in feelings of resignation) are interwoven with findings about limitations of the intervention (eg, limited usefulness due to language barriers) and findings about barriers to effective implementation of the intervention (eg, professional counsellors’ limited time).

**Table 6:** Verbatim qualitative findings about potential adverse effects highlighted in bold

Study report	Findings	Reference to protocol	Location of publicly available protocol
Berger *et al*^60^	The following limitations of the courses were mentioned: language barriers due to the English language of original publications, limited access to publications, too little time for reflection and discussion of the course content, lack of interest of physicians and pharmaceutical industry in personal responsibility of patients, insufficient readiness of patients to overtake responsibility, **feelings of resignation due to complexity**; (…) structured interviews revealed various barriers to implementation; professional counsellors stated they had limited time to undertake systematic search and critical appraisal; they **complained about a lack of evidence-based patient information and decision aids, and the lack of quality standards in counselling**; members of self-help groups reported lacking opportunities to use (evidence-based medicine) the skills; they also **complained about a lack of further support and possibilities to exchange experiences beyond the programme**; some members (n=15) mentioned restrictions due to personal bad health condition; several self-help group members **felt discouraged to use their new skills because they had experienced negative reactions from professionals if they raised critical questions concerning therapeutic issues**	None	Not applicable
Mugisha *et al*^49^	We asked students, teachers, parents and school leaders about any disadvantages of the lessons and looked for potential unintended effects when we observed lessons; some students experienced **undesirable effects related to misapplication of what they learnt in the lessons, misunderstanding of what they learnt and conflicts**	Yes	General repository
Nsangi *et al*^50^	Although teachers found the (informed health choices) lessons enjoyable, some reported having experienced **stress because of teaching something new and it being in addition to their usual subjects**; the majority of teachers and parents expressed **concern about the potential conflicts between themselves and the children resulting from children sometimes challenging their authority, such as asking questions or refusing to take instructions from those in authority**; however, there were no reports of actual conflicts	Yes	Project website
Semakula *et al*^51^	Some participants mentioned that scientific information could potentially be in **conflict with cultural or religious beliefs**; however, no participant reported experiencing these conflicts as a result of listening to the (informed health choices) podcast; we elicited other potential additional effects using the probes in the table; however, no other potential beneficial or adverse effects were reported by participants or observers in the trial	Yes	Project website
Ssenyonga *et al*^52^	We reported the details of potential adverse outcomes associated with this intervention based on the perceptions of participants and our observations in a separate qualitative evidence synthesis of the three trials (reference); the most common potential undesirable effects we found were **partial understanding and misunderstanding of the concepts**	Yes	General repository
Steckelberg *et al*^59^	None of the results indicated that the intervention caused side effects, for example, **excessive demand, risk-taking behaviour, uncertainty and anxiety**	None	Not applicable

Table 7 is an overview of the 16 protocols included in this review. The underlying data include data extracted from those protocols (any mention of potential adverse effects).^29^ Of the 11 protocols for quantitative studies, 8 (73%) mentioned adverse effects in one or more contexts. Only the protocol for the study of the web portal^55^ included plans to assess a specific potential adverse effect, using a specific outcome measure. The protocol for another randomised trial^36^ mentioned plans to develop outcome measures for assessing adverse effects and assessing adverse effects after 1 year. The protocol for yet another randomised trial^35^ mentioned plans to measure adverse outcomes after 1 year, but it was unclear whether the plan was to only measure those outcomes in the intervention arm or in the control arm as well. Other contexts in which protocols for quantitative studies mentioned potential adverse effects included references to associated qualitative studies (process evaluations) exploring experiences of potential adverse effects; plans for safety monitoring (instructing participants to report any adverse events); statements about risks of participation in the study; or references to a risk assessment by an independent body (table 7). All five protocols for qualitative studies mentioned potential adverse effects, all in the context of plans for exploring experiences of such effects.

**Table 7:** Protocols for quantitative and qualitative studies of potential adverse effects.

Methodology	Study report	Protocol mentions potential adverse effects	Context of mention
Quantitative	Austvoll-Dahlgren *et al*^55^	Yes	Plans to assess potential adverse effect
	Chesire *et al*^35^	Yes	Plans for safety monitoring Reference to associated qualitative study exploring experiences of potential adverse effects Plans to measure adverse outcomes in intervention arm or assess potential adverse effects (unclear)
	Cusack *et al*^34^	No	Not applicable
	Mugisha *et al*^37^	Yes	Statement about risk to participants Plans for safety monitoring
	Nsangi *et al*^53^	Yes	Reference to independent risk assessment Plans for safety monitoring Reference to associated qualitative study exploring experiences of potential adverse effects
	Nsangi *et al*^26^	Yes	Reference to independent risk assessment Plans for safety monitoring Reference to associated qualitative study exploring experiences of potential adverse effects
	Okabayashi *et al*^38^	No	Not applicable
	Semakula *et al*^54^	Yes	Reference to independent risk assessment Plans for safety monitoring
	Semakula *et al*^31^	Yes	Reference to independent risk assessment Plans for safety monitoring
	Ssenyonga *et al*^36^	Yes	Plans for safety monitoring Reference to associated qualitative study exploring experiences of potential adverse effects Plans to assess potential adverse effects Statement about risk to participants
	Woloshin *et al*^79^	No	Not applicable
Qualitative	Chesire *et al*^48^	Yes	Plans to explore experiences of potential adverse effects
	Mugisha *et al*^49^	Yes	Plans to explore experiences of potential adverse effects
	Nsangi *et al*^50^	Yes	Plans to explore experiences of potential adverse effects
	Semakula *et al*^51^	Yes	Plans to explore experiences of potential adverse effects
	Ssenyonga *et al*^52^	Yes	Plans to explore experiences of potential adverse effects

## Discussion

### Principal findings

This systematic review shows that researchers—including investigators in this review—generally have not assessed potential adverse effects of interventions to improve critical thinking about health choices. We included 35 reports of quantitative studies of interventions to improve critical thinking about health choices (table 3). In one case (3%), researchers reported that they had assessed a potential adverse effect of the intervention.^55^ In less than one-third of cases, the researchers reported also using qualitative methods (10 of 35, 29%), referred to an associated qualitative study reported elsewhere (9 of 35, 26%) or referred to a publicly available protocol for the quantitative study (11 of 35, 31%). However, in the separate reports of qualitative studies, researchers typically described exploring experiences of potential adverse effects (5 of 7, 71%).^30^ Similarly, when there was a publicly available protocol for the quantitative study, the protocol typically mentioned potential adverse effects in one or more contexts (8 of 11, 73%), although only one protocol included plans specifically for assessing such an effect (table 7). Note that many of the reports of both quantitative and qualitative studies had one or more of the same authors, including investigators in this review.

### Other evidence

As far as we are aware, this is the first systematic review assessing the extent to which researchers have assessed adverse effects of any group of educational interventions. We updated the search for an earlier systematic review by LC *et al*,^12^ which did not address potential harms. We know of three reports of quantitative studies that would have been eligible for inclusion in this review but were published after the search. MO, AF and ADO are co-authors of all three. The reports are of 1-year follow-up assessments of randomised trials of a secondary school intervention in Kenya,^61^ Rwanda^62^ and Uganda.^63^ The reports of the initial assessments are included in this review.^35^,^37^ Neither the reports of the initial or follow-up assessments includes an assessment of any potential adverse effect. However, the report of a meta-analysis of 1-year follow-up data from all three trials includes assessments of adverse outcomes (conflict, stress and acting on misunderstandings), but only in the intervention arms.^64^ Furthermore, MO *et al* have explored experiences of potential adverse effects of the intervention in a qualitative study across the three trial settings.^24^

### Strengths

We updated the extensive search for the original systematic review and supplemented it by incorporating additional study reports included in another, similar systematic review. All together, we increased the sample of study reports included in the original review by a little more than one-third, from 22 to 35 reports. Furthermore, we assessed associated qualitative studies—including an unpublished poster—and protocols for both quantitative and qualitative studies. Two reviewers independently extracted all data for the analysis. The results clearly suggest researchers generally have not assessed potential adverse effects of interventions to improve critical thinking about health choices.

### Limitations

We did not register this review, nor was there a protocol for it. However, we transparently report our methods and rationale, and we have included all data used in the analysis. Thus, there is a low risk of bias due to lack of registration and protocol. Moreover, the original review by LC *et al* was pre-registered with a protocol in PROSPERO (no. CRD42016033103), as was the review by LN *et al* (CRD42015017936). In the search, we did not include terms for adverse effect, since it only would have reduced the number of results. Furthermore, it is unlikely that there are studies in which researchers have only assessed potential adverse effects of relevant interventions. If there were such studies, we likely would have identified them in the search, with the search terms that we did, or in the process of data extraction. We did not search the grey literature for unpublished or non-indexed study reports. Based on the rather unambiguous results of the review, any eligible studies in the grey literature are unlikely to have assessed potential harms. Finally, we did not conduct any meta-analysis or assess potential adverse effects of the interventions, only the extent to which they were included in the studies. Since we did not assess adverse effects, we did not assess risk of bias or rate the certainty of the evidence. Given the dearth of evidence revealed by this review—only one of the quantitative studies included an assessment of a potential adverse effect—a meta-analysis would have been pointless. As noted in the results and earlier in the discussion, there was overlap between authors of this review and some of the included studies. Two reviewers independently extracted all data for the review, and we have made all the data underlying the review available in a public repository. Most of the authors of the review, including authors who contributed to data extraction, were not authors of any of the included studies.

### Implications

Researchers should pay more attention to potential adverse effects of interventions to improve critical thinking about health choices. In protocols for studies of such interventions, they should report the potential adverse effects that they plan to assess specifically, as well as why and how. They should also report how they plan to assess potential adverse effects of the intervention in general, since the intervention might have adverse effects that they considered, but decided not to assess, or adverse effects that they failed to consider. If they do not plan to assess potential adverse effects at all, they should explain why. Furthermore, they should address any need for qualitative studies to complement the assessments or mitigate the lack thereof. In the study reports, authors should describe relevant results or explain relevant deviations from the protocol. If the study did not assess any potential adverse effect, the authors should explicitly address this non-inclusion in the methods section and discussion. Policymakers and educators making decisions about implementing relevant interventions should consider the lack of evidence about potential adverse effects. The findings of this review suggest a need for studies that facilitate assessing potential adverse effects of relevant interventions, for example, development and evaluation of outcome measures. MO *et al* have developed^65^ and later applied and revised^24^ a framework of potential adverse effects of interventions to improve critical thinking about health, which can be used as a starting point. The framework includes six overarching categories: decision-making harms, psychological harms, equity harms, group and social harms, waste and other harms. We used the framework to develop outcome measures used for the meta-analysis after 1 year.^64^ However, the measures require adaption and testing for use in assessing effects (measuring and comparing outcomes across intervention and control arms).

## Supplementary material

10.1136/bmjopen-2025-108268 online supplemental file 1

10.1136/bmjopen-2025-108268 online supplemental file 2

10.1136/bmjopen-2025-108268 online supplemental file 3

10.1136/bmjopen-2025-108268 online supplemental file 4

## Data Availability

Data are available in a public, open access repository.
