# Supplementary material for: Do studies of interventions to improve laypeople’s critical thinking about health choices assess potential harms? A systematic review
Source: BMJ Open. 2026 Apr 24;16(4):e108268. doi: 10.1136/bmjopen-2025-108268 (PMC13110562; doi:10.1136/bmjopen-2025-108268)
Supplement: online supplemental file 3 [file bmjopen-16-4-s003.docx]

**Supplementary File 3**

**Detailed search strategies (second search update)**

**ERIC via Ovid**

ERIC <1965 to December 2024>

| **#** | **Query** | **Results from 13 Feb 2025** |
| --- | --- | --- |
| 1 | (Instruction or Training or Workshop* or Course*).af. | 742,308 |
| 2 | (Evidence?based or Critical* apprais* or Health risk* or Health* information or Health literacy or Medical information or Health* advice or Medical advice or Health* research or Medical research or Statistics or Causality or Controlled trial* or Clinical trial* or Fair test or Systematic review or Health choice* or Health decision*).af. | 83,545 |
| 3 | (Understand* or Competen* or Apprais* or Skill* or Knowledge or Evaluat* or Health literacy).af. | 917,788 |
| 4 | (Investigat* or Compar* or Studie* or Study or Observ*).af. | 1,098,075 |
| 5 | ((Instruction or Training or Workshop* or Course*) and (Evidence?based or Critical* apprais* or Health risk* or Health* information or Health literacy or Medical information or Health* advice or Medical advice or Health* research or Medical research or Statistics or Causality or Controlled trial* or Clinical trial* or Fair test or Systematic review or Health choice* or Health decision*) and (Understand* or Competen* or Apprais* or Skill* or Knowledge or Evaluat* or Health literacy) and (Investigat* or Compar* or Studie* or Study or Observ*)).af. | 12,427 |
| 6 | limit 5 to yr="2023 - 2025" | 1,393 |

**Web of Science**

(((((TS=(People OR Patient* OR “School children” OR Student* OR Public OR Consumer* OR Participant*)) AND TS=(Education* OR Teaching OR Taught OR Train* OR Workshop* OR Course*)) AND TS=(((“Evidence?based” OR “Critical* apprais*” OR “Health risk*” OR “Health* information” OR “Health literacy” OR “Medical information” OR “Health* advice” OR “Medical advice” OR “Health* research” OR “Medical research” OR Statistics OR “Controlled trial*” OR “Clinical trial*” OR RCT* OR “Fair test” OR “Systematic review*” OR “Health choice*” OR “Health decision*”) NEAR/2 (Understand* OR Competen* OR Apprais* OR Skill* OR Knowledge OR Evaluat*)))) AND TS=((“Controlled Trial” OR “Clinical Trial” OR “Multi$cent* Study” OR “Program Evaluation” OR ((Random*) NEAR/2 group*) OR “Random allocation” OR “Control group” or “Intervention group” OR “Comparison group” OR ((Prospective OR Crossover) NEAR/2 (Study OR Studies or Design)) OR (Before NEAR/2 After) OR (Pre NEAR/2 Post) OR Quasi?experimental OR “Time series” OR “Time point*” OR “Repeated measure*” OR “Observational study”))) NOT ALL=(“Conference abstract” OR “Conference paper” OR “Conference review” OR “Editorial Item” OR “Erratum Item”)) NOT TI=((“Systematic review” OR “Literature review”))

**Publication date** 2023-01-01 to 2025-02-13

**Results:** 645

**Embase via Ovid**

Embase Classic+Embase <1947 to 2025 February 12>

| **#** | **Query** | **Results from 13 Feb 2025** |
| --- | --- | --- |
| 1 | exp miscellaneous named groups/ or exp patients/ or (People or Patient* or School or Children or Student* or Public or Consumer* or Participant*).tw. | 17,479,008 |
| 2 | exp health education/ or exp teaching/ or Education.tw. or Educational.tw. or Teaching.tw. or Taught.tw. or Train.tw. or Trained.tw. or Training.tw. or Workshop.tw. or Workshops.tw. | 2,406,711 |
| 3 | (exp evidence based practice/ or exp information literacy/) and (Understand* or Competen* or Apprais* or Skill* or Evaluat* or Knowledge).tw. | 835,656 |
| 4 | 1 and 2 and 3 | 103,699 |
| 5 | ((Evidence?based or Critical* apprais* or Health risk* or Random* or Control group or Comparison group or Health* information or Health literacy or Medical information or Health* advice or Medical advice or Health* research or Medical research or Statistics or Controlled trial* or Clinical trial* or RCT* or Systematic review*) adj2 (Understand* or Competen* or Apprais* or Skill* or Knowledge or Evaluat* or Assess* or Think*)).tw. | 139,373 |
| 6 | 4 or 5 | 235,603 |
| 7 | (Randomized Controlled Trial or Controlled Clinical Trial or Multicenter Study).pt. or exp Epidemiologic Studies/ or Program Evaluation/ or Pilot Projects/ or Random*.tw. or (Group* adj2 (Random* or Between or Control or Intervention)).ab. or Controlled trial.tw. or (Intervention* or Controlled or Control group or Compare* or Comparison* or ((Prospectiv* or Crossover) adj2 (Study or Studies or Design)) or (Before adj2 After) or (Pre adj2 Post) or Pre?test or Post?test or Quasi?experiment* or Time series or Evaluat* or Effectiveness or Impact or Time series or Time point or Repeated measure*).tw. or Observational.tw. or Observe.tw. or Observation.tw. or Observing.tw. or Survey.tw. or Surveys.tw. or Surveyed.tw. or Interviews.tw. or Comparison.tw. | 20,273,047 |
| 8 | 6 and 7 | 216,827 |
| 9 | exp health care personnel/ or exp vocational education/ or exp health student/ | 2,236,187 |
| 10 | 8 not 9 | 177,993 |
| 11 | (Review or Meta analysis or News or Comment or Editorial or Letter).pt. or cochrane database of systematic reviews.jn. or comment on.mp. or (systematic review or literature review).ti. [mp=title, abstract, heading word, drug trade name, original title, device manufacturer, drug manufacturer, device trade name, keyword heading word, floating subheading word, candidate term word] | 5,732,886 |
| 12 | 10 not 11 | 127,073 |
| 13 | exp Animals/ not (Animals/ and Humans/) | 34,712,529 |
| 14 | 12 not 13 | 6,632 |
| 15 | (202212* or 2023* or 2024* or 2025*).dc,dd,yr. | 4,587,688 |
| 16 | 14 and 15 | 626 |

**MEDLINE via Ovid**

2022-January 29,2025

| 1 | exp Persons/ or exp Patients/ or (People or Patient or Patients or People or School or children or Student or Students or Public or Consumer or Consumers or Participant or Participants).tw. | 16411162 |
| --- | --- | --- |
| 2 | exp Health Education/ or exp Teaching/ or Education.tw. or Educational.tw. or Teaching.tw. or Taught.tw. or Train.tw. or Trained.tw. or Training.tw. or Workshop.tw. or Workshops.tw. | 1797402 |
| 3 | (exp Evidence-Based practice/ or exp Information literacy/) and (Understand or Understanding or Competency or Competencies or Appraise or Appraisal or Skill or Skills or Evaluate or Evaluation or Knowledge).tw. | 32744 |
| 4 | 1 and 2 and 3 | 13100 |
| 5 | ((Evidence based or Evidence-based or Critical or Critically or Health risk or Health risks or Randomisation or Randomization or Control group or Comparison group or Health information or Health literacy or Health information or Healthcare information or Medical information or Health advice or Healthcare advice or Medical advice or Health research or Healthcare research or Medical research or Statistics or Controlled trial or Controlled trial or Clinical trial or Clinical trials or RCT or RCTs or Systematic review or Systematic reviews) adj2 (Understand or Understanding or Competency or Competencies or Appraise or Appraisal or Skill or Skills or Knowledge or Evaluate or Evaluating or Evaluation or Assess or Assessing or Think or Thinking)).tw. | 99425 |
| 6 | 4 or 5 | 110013 |
| 7 | (Randomized Controlled Trial or Controlled Clinical Trial or Multicenter Study).pt. or exp Epidemiologic Studies/ or Program Evaluation/ or Pilot Projects/ or (Randomised or Randomized or Randomise or Randomize or Randomly or Random allocation).tw. or ((Group or Groups) adj2 (random* or between or control or intervention)).ab. or Controlled trial.tw. or (intervention* or controlled or control group or compare or comparison* or compared or ((prospectiv* or crossover) adj2 (study or studies or design)) or (before adj2 after) or (pre adj2 post) or pretest or pre test or posttest or post test or quasiexperiment* or time series or quasi experiment* or evaluat* or effectiveness or impact or time series or time point? or repeated measur*).tw. or Observational.tw. or Observe.tw. or Observation.tw. or Observing.tw. or Survey.tw. or Surveys.tw. or Surveyed.tw. or Interviews.tw. or Comparison.tw. | 14585931 |
| 8 | 6 and 7 | 88902 |
| 9 | exp Health Personnel/ or exp Education, Professional/ or exp Students, Health Occupations/ | 952526 |
| 10 | 8 not 9 | 79494 |
| 11 | (Review or Meta Analysis or News or Comment or Editorial or Letter).pt. or cochrane database of systematic reviews.jn. or comment on.cm. or (systematic review or literature review).ti. | 6163018 |
| 12 | 10 not 11 | 47174 |
| 13 | exp Animals/ not (Animals/ and Humans/) | 25479860 |
| 14 | 12 not 13 | 12879 |
| 15 | limit 14 to yr="2022 -Current" | 4768 |

**CENTRAL**

2022 up to January 30, 2025

| #1 | [mh Patients] OR (People OR Patient OR Patients OR People OR Schoolchildren OR Student OR Students OR Public OR Consumer OR Consumers OR Participant OR Participants):ti,ab,kw | 1553703 |
| --- | --- | --- |
| #2 | [mh Teaching] OR Education:ti,ab OR Educational:ti,ab OR Teaching:ti,ab OR Taught:ti,ab OR Train:ti,ab OR Trained:ti,ab OR Training:ti,ab OR Workshop:ti,ab OR Workshops:ti,ab,kw | 239101 |
| #3 | ([mh "Evidence-Based practice"] OR [mh "Information literacy"]) AND (Understand OR Understanding OR Competency OR Competencies OR Appraise OR Appraisal OR Skill OR Skills OR Evaluate OR Evaluation OR Knowledge):ti,ab,kw | 1998 |
| #4 | #1 AND #2 AND #3 | 709 |
| #5 | (("Evidence based" OR Evidence-based OR Critical OR Critically OR "Health risk" OR "Health risks" OR Randomisation OR Randomization OR "Control group" OR "Comparison group" OR "Health information" OR "Health literacy" OR "Health information" OR "Healthcare information" OR "Medical information" OR "Health advice" OR "Healthcare advice" OR "Medical advice" OR "Health research" OR "Healthcare research" OR "Medical research" OR Statistics OR "Controlled trial" OR "Controlled trial" OR "Clinical trial" OR "Clinical trials" OR RCT OR RCTs OR "Systematic review" OR "Systematic reviews") NEXT/2 (Understand OR Understanding OR Competency OR Competencies OR Appraise OR Appraisal OR Skill OR Skills OR Knowledge OR Evaluate OR Evaluating OR Evaluation OR Assess OR Assessing OR Think OR Thinking)):ti,ab,kw | 35686 |
| #6 | #4 OR #5 | 36287 |
| #7 | [mh "Health Personnel"] OR [mh "Students, Health Occupations"] | 19051 |
| #8 | #6 NOT #7 | 35309 |
| #9 | with Publication Year from 2022 to present , in Trials | 8825 |

**CINAHL**

01/01/2022- 31/03/2025

| 1 | (MH "Named Groups+") OR (MH "Patients+") OR (TX (People OR Patient OR Patients OR People OR Schoolchildren OR Student OR Students OR Public OR Consumer OR Consumers OR Participant OR Participants)) | 6.608.499 |
| --- | --- | --- |
| 2 | (MH "Health Education+") OR (MH "Teaching+") OR (TX Education OR TX Educational OR TX Teaching OR TX Education OR TX Educational OR TX Teaching OR TX Taught OR TX Train OR TX Trained OR TX Training OR TX Workshop OR TX Workshops) | 2.266.583 |
| 3 | (MH "Professional Practice, Evidence-Based" OR MH "Information Literacy") AND (TX (Understand OR Understanding OR Competency OR Competencies OR Appraise OR Appraisal OR Skill OR Skills OR Evaluate OR Evaluation OR Knowledge)) | 17.423 |
| 4 | 1 AND 2 AND 3 | 10.411 |
| 5 | TX (("Evidence based" OR Evidence-based OR Critical OR Critically OR "Health risk" OR "Health risks" OR Randomisation OR Randomization OR "Control group" OR "Comparison group" OR "Health information" OR "Health literacy" OR "Health information" OR "Healthcare information" OR "Medical information" OR "Health advice" OR "Healthcare advice" OR "Medical advice" OR "Health research" OR " Healthcare research" OR "Medical research" OR Statistics OR "Controlled trial" OR "Controlled trial" OR "Clinical trial" OR "Clinical trials" OR RCT OR RCTs OR "Systematic review" OR "Systematic reviews") W2 (Understand OR Understanding OR Competency OR Competencies OR Appraise OR Appraisal OR Skill OR Skills OR Knowledge OR Evaluate OR Evaluating OR Evaluation OR Assess OR Assessing OR Think OR Thinking)) | 124.063 |
| 6 | 4 OR 5 | 132.126 |
| 7 | (PT ("Randomized Controlled Trial" OR "Controlled Clinical Trial" OR "Multicenter Study")) OR (MH "Epidemiological Research+") OR (MH "Program Evaluation") OR (MH "Pilot Studies") OR (TX (Randomised OR Randomized OR Randomise OR Randomize OR Randomly OR "Random allocation")) OR (AB ((Group OR Groups) W2 (random* OR between OR control OR intervention))) OR (TX "Controlled trial") OR (TX (intervention* OR controlled OR control group OR compare OR comparison* OR compared OR ((prospectiv* OR crossover) W2 (study OR studies OR design)) OR (before W2 after) OR (pre W2 post) OR pretest OR pre test OR posttest OR "post test" OR quasiexperiment* OR "time series" OR quasi experiment* OR evaluat* OR effectiveness OR impact OR "time series" OR time point? OR repeated measur*)) | 4.125.307 |
| 8 | 6 AND 7 | 120.555 |
| 9 | (MH "Health Personnel+") OR (MH "Refresher Courses") OR (MH "Students, Health Occupations+") | 759.294 |
| 10 | 8 NOT 9 | 13.308 |
| 11 | (PT (Review OR "Meta Analysis" OR News OR Comment OR Editorial OR Letter)) OR (JN "cochrane database of systematic reviews") OR (CR comment on) OR (TI ("systematic review" OR "literature review")) | 94.147 |
| 12 | 10 NOT 11 | 79.192 |
| 13 | Filtro: 31/12/2022 - 30/03/2025 | 9607 |

**Epistemonikos**

2022 up to January 30, 2025

| #1 | (education* OR teach* OR taught* OR train OR trained OR training OR workshop*) AND (("health choices" OR "evidence based" OR "evidence-based" OR Critical OR Critically OR "Health risk" OR "Health risks" OR Randomisation OR Randomization OR "Control group" OR "Comparison group" OR "Health information" OR "Health literacy" OR "Health information" OR "Healthcare information" OR "Medical information" OR "Health advice" OR "Healthcare advice" OR "Medical advice" OR "Health research" OR "Healthcare research" OR "Medical research" OR Statistics OR "Controlled trial" OR "Controlled trial" OR "Clinical trial" OR "Clinical trials" OR RCT OR RCTs OR "Systematic review" OR "Systematic reviews") AND (understand* OR competenc* OR appraise OR appraisal OR skill OR skills OR evaluate OR evaluation OR knowledge)) |
| --- | --- |
| #2 | (persons* OR patient* OR people* OR school* OR children OR student* OR public OR consumer* OR participant OR participants) |
| #3 | #1 AND #2 |
| Total hits | 6,155 |
