# Supplementary material for: Do studies of interventions to improve laypeople’s critical thinking about health choices assess potential harms? A systematic review
Source: BMJ Open. 2026 Apr 24;16(4):e108268. doi: 10.1136/bmjopen-2025-108268 (PMC13110562; doi:10.1136/bmjopen-2025-108268)
Supplement: online supplemental file 4 [file bmjopen-16-4-s004.docx]

# **SUPLEMENTARY FILE 4**

# **EXCLUDED ARTICLES**

|  | **Reference** | **Reason for exclusion** |
| --- | --- | --- |
|  | Abu-Gharbieh, Eman, Khalidi, Doaa Al, Baig, Mirza R, Khan, Saeed A. Refining knowledge, attitude and practice of evidence-based medicine (EBM) among pharmacy students for professional challenges. Saudi pharmaceutical journal : SPJ : the official publication of the Saudi Pharmaceutical Society. 2015;23(2):162-6. [e0f9eb44e1128682d0f78d9b23406b500c0a61cf] | Not lay people |
|  | Basheer, Aneesh, Iqbal, Nayyar, Prabakaran, Stalin, Simiyon, Manjula, Anandan, Velavan. Simulated Randomized Controlled Trial to Learn Critical Appraisal (SiRCA): A Randomized Controlled Study of Effectiveness Among Undergraduate Medical Students. Cureus. 2021;13(11):e19946. [7e7c702d70bbc760c098488c78493215ef192b18] | Not lay people |
|  | Gajbhiye, Snehalata, Tripathi, Raakhi, Parmar, Urwashi, Khatri, Nishtha, Potey, Anirudha. Critical appraisal of published research papers - A reinforcing tool for research methodology: Questionnaire-based study. Perspectives in clinical research. 2021;12(2):100-105. [48a47c2c295b4e9d436410852b6c00051eaedc1b] | Not lay people |
|  | Goodarzi, H, Teymourzadeh, E, Rahimi, S, Nasiri, T. Efficacy of active and passive evidence-based practice training for postgraduate medical residents: a non-randomized controlled trial. 2021;14(1):317. [4231f716c1c6d512329e6905485aa414b56c158a] | Not lay people |
|  | Jelsness-Jørgensen, Lars-Petter. Does a 3-week critical research appraisal course affect how students perceive their appraisal skills and the relevance of research for clinical practice? A repeated cross-sectional survey. Nurse Education Today. 2015;35(1):e1-5. [f15001845201f063b630363c9e2ac1d20966cd08] | Not lay people |
|  | Krnic Martinic M, Čivljak M, Marušić A, Sapunar D, Poklepović Peričić T, Buljan I, Tokalić R, Mališa S, Neuberg M, Ivanišević K, Aranza D, Skitarelić N, Zoranić S, Mikšić Š, Čavić D, Puljak L. Web-Based Educational Intervention to Improve Knowledge of Systematic Reviews Among Health Science Professionals: Randomized Controlled Trial. Journal of medical Internet research. 2022;24(8):e37000. [a05c33ff635af38ab5a88838d2d4c5f4ce900def] | Not lay people |
|  | Najafi, Naghmeh Feyzi, Asgari, Imaneh. Effectiveness of a program on evidence-based dentistry in dental students. Journal of education and health promotion. 2017;6(101593794):34. [0e2b969f0d92992e303f89d88610682fdd559312] | Not lay people |
|  | Nelson, B, Ingard, C, Nelson, D. Teaching trainees how to critically evaluate the literature - a crossover study at two pediatric residency programs. 2017;8(pp 137‐141). [820b7b7b4ea462d4116e4d5da67aafd255773c7d] | Not lay people |
|  | Sasannia, Sarvin, Amini, Mitra, Moosavi, Mahsa, Askarinejad, Amir, Moghadami, Mana, Ziaee, Hasti, Vara, Fatemeh. Critical appraisal skills training to undergraduate medical students: A Randomized Control Study. Journal of advances in medical education & professionalism. 2022;10(4):253-258. [ed4da0b9bd031e8ca7e245b79e4f4d1fddf7264f] | Not lay people |
|  | Schneider, Michael, Evans, Roni, Haas, Mitchell, Leach, Matthew, Delagran, Louise, Hawk, Cheryl, Long, Cynthia, Cramer, Gregory D, Walters, Oakland, Vihstadt, Corrie, Terhorst, Lauren. The effectiveness and feasibility of an online educational program for improving evidence-based practice literacy: an exploratory randomized study of US chiropractors. Chiropractic & manual therapies. 2016;24(101551481):27. [58f22f75fd5adda1d44e7662b33708307a0cc08a] | Not lay people |
|  | Wapau H, Kris E, Roeder L, McDonald M. Community-driven health research in the Torres Strait. Australian journal of primary health. 2022; [1b4f241cfb5da00e3d9a102119ff376450e4a04d] | Not lay people |
|  | Wenke RJ, Thomas R, Hughes I, Mickan S. The effectiveness and feasibility of TREAT (Tailoring Research Evidence and Theory) journal clubs in allied health: a randomised controlled trial. BMC medical education. 2018;18(1):104. [253d27f3a16f6c86d0a2046ebd7edf201a5464f7] | Not lay people |
|  | Maloney, LM, Marshall, RT, Werfel, PA, Johnson, SE. Using a Journal Club Series to Introduce Paramedic Students to Research Fundamentals and Critical Appraisal of Medical Literature. PREHOSPITAL AND DISASTER MEDICINE. 2019;34(4):449-453.[47cc8b704d07a5a2d91460d1fed51edb4ede5a81] | Not lay people |
|  | Paravattil, B, Shabana, S, Rainkie, D, Wilby, KJ. Evaluating knowledge, skills, and practice change after an accredited evidence-based medicine course for community pharmacy preceptors. CURRENTS IN PHARMACY TEACHING AND LEARNING. 2019;11(8):802-809. [8ecba482384d4f9d8003df665f94dd6cf376d07e] | Not lay people |
|  | Ajuwon, Grace Ada, Ajuwon, Ademola Johnson. Teaching high school students to use online consumer health resources on mobile phones: outcome of a pilot project in Oyo State, Nigeria. Journal of the Medical Library Association. 2019;107(2):194-202. [db1a15f1c3f67ffa2f1e224eddfb1508dbe62130] | No IHC Key Concepts |
|  | Bay, Jacquie L, Vickers, Mark H, Mora, Helen A, Sloboda, Deborah M, Morton, Susan M. Adolescents as agents of healthful change through scientific literacy development: A school-university partnership program in New Zealand. International journal of STEM education. 2017;4(1):15. [f3974728c97a088e2d8565441bba1e4d430eb00a] | No IHC Key Concepts |
|  | Cardoso, Mário, Baixinho, Cristina Lavareda, Ferreira, Óscar, Nascimento, Patricia, Pedrosa, Rita, Gonçalves, Paulo. Learning evidence based practice through involvement in investigation activities - the self-perception of students. Cogit. Enferm. 2021;26:e79806-e79806. [08e4447de8430eacfb780fd46557ecaeb3524419] | No IHC Key Concepts |
|  | Chesire, Faith, Ochieng, Marlyn, Mugisha, Michael, Ssenyonga, Ronald, Oxman, Matt, Nsangi, Allen, Semakula, Daniel, Nyirazinyoye, Laetitia, Lewin, Simon, Sewankambo, Nelson K, Kaseje, Margaret, Oxman, Andrew D, Rosenbaum, Sarah. Contextualizing critical thinking about health using digital technology in secondary schools in Kenya: a qualitative analysis. Pilot and feasibility studies. 2022;8(1):227. [ba7ce9946b7cce962182b1840e3a5919ed5586ee] | No IHC Key Concepts |
|  | Cook, David A., Lineberry, Matthew. Consequences validity evidence: Evaluating the impact of educational assessments. Academic Medicine. 2016;91(6). [dff0398816154b89a830296c4b67bdd2a179be2e] | No IHC Key Concepts |
|  | Davies C, McGillion M, Rowland C, Matthews D. Can inferencing be trained in preschoolers using shared book-reading? A randomised controlled trial of parents' inference-eliciting questions on oral inferencing ability. Journal of child language. 2020;47(3):655-679. [5cf4423b486b42cd1b154fa7c684e6aa40124ca3] | No IHC Key Concepts |
|  | De Main, Atami S., Xie, Bo, Shiroma, Kristina, Yeh, Tom, Davis, Nathan, Han, Xu. Assessing the Effects of eHealth Tutorials on Older Adults' eHealth Literacy. Journal of Applied Gerontology. 2022;41(7):1675-1685. [e14aea193f7228b782ed5c785337a640cc0b39b3] | No IHC Key Concepts |
|  | El Miedany, Y, El Gaafary, M, Lotfy, H, El Aroussy, N, Mekkawy, D, Nasef, SI, Farag, Y, Almedany, S, Wassif, G, PRINTO, Egypt. Shared decision-making aid for juvenile idiopathic arthritis: moving from informative patient education to interactive critical thinking. Clinical rheumatology. 2019;38(11):3217‐3225. [33ff4078b38fe6ba1ec27ce89aa1feb8cd00d7fe] | No IHC Key Concepts |
|  | Elsharkawy NB, Abdelaziz EM, Ouda MM, Oraby FA. Effectiveness of Health Information Package Program on Knowledge and Compliance among Pregnant Women with Anemia: A Randomized Controlled Trial. International journal of environmental research and public health. 2022;19(5). [ed91c7e21efa252aa90cfd0602f8d92651259e1b] | No IHC Key Concepts |
|  | Golembiewski EH, Mainous AG, Rahmanian KP, Brumback B, Rooks BJ, Krieger JL, Goodman KW, Moseley RE, Harle CA. An Electronic Tool to Support Patient-Centered Broad Consent: A Multi-Arm Randomized Clinical Trial in Family Medicine. Annals of family medicine. 2021;19(1):16-23. [f2cab199a7f2d977eacd74247754a506d2cd7a2d] | No IHC Key Concepts |
|  | Housten AJ, Kamath GR, Bevers TB, Cantor SB, Dixon N, Hite A, Kallen MA, Leal VB, Li L, Volk RJ. Does Animation Improve Comprehension of Risk Information in Patients with Low Health Literacy? A Randomized Trial. Medical decision making : an international journal of the Society for Medical Decision Making. 2020;40(1):17-28. [175aea0abfb881fdd10f1c1cabde8ecaee371807] | No IHC Key Concepts |
|  | Imms C, Novak I, Kerr C, Shields N, Randall M, Harvey A, Graham HK, Reddihough D. Improving allied health professionals' research implementation behaviours for children with cerebral palsy: protocol for a before-after study. Implementation science : IS. 2015;10:16. [c532764c128bea635d41fa91abec2b1d62ec8318] | No IHC Key Concepts |
|  | Isselhard A, Töpper M, Berger-Höger B, Steckelberg A, Fischer H, Vitinius F, Beifus K, Köberlein-Neu J, Wiedemann R, Rhiem K, Schmutzler R, Stock S. Implementation and evaluation of a nurse-led decision-coaching program for healthy breast cancer susceptibility gene (BRCA1/2) mutation carriers: a study protocol for the randomized controlled EDCP-BRCA study. Trials. 2020;21(1):501. [9cab08c0a2d3e3282b0ca13327d5f35e6f1d9c8b] | No IHC Key Concepts |
|  | Itoh N, Mishima H, Yoshida Y, Yoshida M, Oka H, Matsudaira K. Evaluation of the Effect of Patient Education and Strengthening Exercise Therapy Using a Mobile Messaging App on Work Productivity in Japanese Patients With Chronic Low Back Pain: Open-Label, Randomized, Parallel-Group Trial. JMIR mHealth and uHealth. 2022;10(5):e35867. [df6a75438efead41f5fd9263dcb2e2e55dd84526] | No IHC Key Concepts |
|  | Jaensson M, Stenberg E, Liang Y, Nilsson U, Dahlberg K. Validity and reliability of the Swedish Functional Health Literacy scale and the Swedish Communicative and Critical Health Literacy scale in patients undergoing bariatric surgery in Sweden: a prospective psychometric evaluation study. BMJ open. 2021;11(11):e056592. [cd32b4a224bdc4c388a4abfb781c7363630b4009] | No IHC Key Concepts |
|  | Kludas L, Kriston L, Metzner F, Wichmann M, Pawils S. [Effects of written educational material on parental health knowledge depending on socioeconomic status : A randomized controlled trial]. Bundesgesundheitsblatt, Gesundheitsforschung, Gesundheitsschutz. 2018;61(4):385-393. [e8df4df800eaa8db2b9c35e495ad1bafe0567e97] | No IHC Key Concepts |
|  | Linn, AJ, van Dijk, L, van Weert, JCM, Gebeyehu, BG, van Bodegraven, AA, Smit, EG. Creating a synergy effect: a cluster randomized controlled trial testing the effect of a tailored multimedia intervention on patient outcomes. Patient education and counseling. 2018;101(8):1419‐1426. [9367d5a8a7357d436543b724abd17f7ed6084ca1] | No IHC Key Concepts |
|  | Luis J. Rodríguez Muñiz, Laura Muñiz-Rodríguez, Claudia Vásquez, Ángel Alsina i Pastells. ¿Cómo promover la alfabetización estadística y de datos en contexto?: Estrategias y recursos a partir de la COVID-19 para Educación Secundaria. 2020;(104). [c21d638daf77a860fd292869b9e89978bdf380f5] | No IHC Key Concepts |
|  | McCaffery KJ, Morony S, Muscat DM, Smith SK, Shepherd HL, Dhillon HM, Hayen A, Luxford K, Meshreky W, Comings J, Nutbeam D. Evaluation of an Australian health literacy training program for socially disadvantaged adults attending basic education classes: study protocol for a cluster randomised controlled trial. BMC public health. 2016;16(1):454. [e484555375c5ced9851d429db58c396990bd94fb] | No IHC Key Concepts |
|  | McElfish PA, Purvis RS, Scott AJ, Haggard-Duff LK, Riklon S, Long CR. "The results are encouragements to make positive changes to be healthier:" qualitative evaluation of Marshallese participants' perceptions when receiving study results in a randomized control trial. Contemporary clinical trials communications. 2020;17:100543. [0b7605a7be7d6090b82113f177242b2af877fe24] | No IHC Key Concepts |
|  | Morony S, Lamph E, Muscat D, Nutbeam D, Dhillon HM, Shepherd H, Smith S, Khan A, Osborne J, Meshreky W, Luxford K, Hayen A, McCaffery KJ. Improving health literacy through adult basic education in Australia. Health promotion international. 2018;33(5):867-877. [cfb1a5bd834940a20e73c8924c7eb5112255aa73] | No IHC Key Concepts |
|  | Muscat, Danielle M, Morony, Suzanne, Trevena, Lyndal, Hayen, Andrew, Shepherd, Heather L, Smith, Sian K, Dhillon, Haryana M, Luxford, Karen, Nutbeam, Don, McCaffery, Kirsten J. Skills for Shared Decision-Making: Evaluation of a Health Literacy Program for Consumers with Lower Literacy Levels. Health literacy research and practice. 2019;3(3 Suppl):S58-S74. [5b64b2cb69ae9b9aeef322a36c314b8388158c4b] | No IHC Key Concepts |
|  | Nsangi A, Semakula D, Glenton C, Lewin S, Oxman AD, Oxman M, Rosenbaum S, Dahlgren A, Nyirazinyoye L, Kaseje M, Rose CJ, Fretheim A, Sewankambo NK. Informed health choices intervention to teach primary school children in low-income countries to assess claims about treatment effects: process evaluation. BMJ open. 2019;9(9):e030787. [8b4ae7e7c234c4e5a665df85d2df450dac9df00c] | No IHC Key Concepts |
|  | Nsangi, Allen, Semakula, Daniel, Rosenbaum, Sarah E, Oxman, Andrew David, Oxman, Matt, Morelli, Angela, Austvoll-Dahlgren, Astrid, Kaseje, Margaret, Mugisha, Michael, Uwitonze, Anne-Marie, Glenton, Claire, Lewin, Simon, Fretheim, Atle, Sewankambo, Nelson Kaulukusi. Development of the informed health choices resources in four countries to teach primary school children to assess claims about treatment effects: a qualitative study employing a user-centred approach. Pilot and feasibility studies. 2020;6(101676536):18. [b8eff13d050ddb3ac9ffc1320d5c9128b34f8dfa] | No IHC Key Concepts |
|  | Politi MC, Kuzemchak MD, Kaphingst KA, Perkins H, Liu J, Byrne MM. Decision Aids Can Support Cancer Clinical Trials Decisions: Results of a Randomized Trial. The oncologist. 2016;21(12):1461-1470. [c1a0ff55a0fe5db8cef626f433a438e602932f12] | No IHC Key Concepts |
|  | Rahn, AC, Backhus, I, Fuest, F, Riemann-Lorenz, K, Köpke, S, van de Roemer, A, Mühlhauser, I, Heesen, C. Comprehension of confidence intervals - development and piloting of patient information materials for people with multiple sclerosis: qualitative study and pilot randomised controlled trial. BMC medical informatics and decision making. 2016;16(1):122. [57e1d53c6c52d72180ca7bc6461849a71c65eb9d] | No IHC Key Concepts |
|  | Rusmansyah, M. Isra’i Rahman, Almubarakand, Masniah. Training of Scientific Literation and Self Efficacy Students Using Scientific Critical Thinking (SCT) Models. 2021;1788(1). [d898af58f466da8d2d5868d8d92317bb9fc94baf] | No IHC Key Concepts |
|  | Sanders, Mechelle, Bringley, Kate, Thomas, Marie, Boyd, Michele, Farah, Subrina, Fiscella, Kevin. Promoting MedlinePlus utilization in a federally qualified health center using a multimodal approach. Journal of the Medical Library Association. 2018;106(3):361-369. [f40d72516601f85328d72c0ab21c0fabbae0f5c7] | No IHC Key Concepts |
|  | Schiefer J, Golle J, Tibus M, Herbein E, Gindele V, Trautwein U, Oschatz K. Effects of an extracurricular science intervention on elementary school children's epistemic beliefs: A randomized controlled trial. The British journal of educational psychology. 2020;90(2):382-402. [a8ec311cf200f4a0c1df534d51e2418376f3e4fe] | No IHC Key Concepts |
|  | Semakula, Daniel, Nsangi, Allen, Oxman, Matt, Rosenbaum, Sarah Ellen, Oxman, Andrew David, Austvoll-Dahlgren, Astrid, Glenton, Claire, Lewin, Simon, Kaseje, Margaret, Morelli, Angela, Fretheim, Atle, Sewankambo, Nelson Kaulukusi. Development of mass media resources to improve the ability of parents of primary school children in Uganda to assess the trustworthiness of claims about the effects of treatments: a human-centred design approach. Pilot and feasibility studies. 2019;5(101676536):155. [e0d3d69e1f7b135695491e46facff19d0e446a9d] | No IHC Key Concepts |
|  | Shinde S, Weiss HA, Khandeparkar P, Pereira B, Sharma A, Gupta R, Ross DA, Patton G, Patel V. A multicomponent secondary school health promotion intervention and adolescent health: An extension of the SEHER cluster randomised controlled trial in Bihar, India. PLoS medicine. 2020;17(2):e1003021. [7ea680c54020d8b4deb4ddc41966583dbfc10103] | No IHC Key Concepts |
|  | Smith CA, Chang E, Gallego G, Khan A, Armour M, Balneaves LG. An education intervention to improve decision making and health literacy among older Australians: a randomised controlled trial. BMC geriatrics. 2019;19(1):129. [887a3651a165adcb8a66bb717fc293bba6a17a6f] | No IHC Key Concepts |
|  | Ssenyonga R, Sewankambo NK, Mugagga SK, Nakyejwe E, Chesire F, Mugisha M, Nsangi A, Semakula D, Oxman M, Nyirazinyoye L, Lewin S, Kaseje M, Oxman AD, Rosenbaum S. Learning to think critically about health using digital technology in Ugandan lower secondary schools: A contextual analysis. PloS one. 2022;17(2):e0260367. [f955590c18163aa8208b73a45a991f01770cc314] | No IHC Key Concepts |
|  | Thompson CA, Taber JM, Sidney PG, Fitzsimmons CJ, Mielicki MK, Matthews PG, Schemmel EA, Simonovic N, Foust JL, Aurora P, Disabato DJ, Seah THS, Schiller LK, Coifman KG. Math matters: A novel, brief educational intervention decreases whole number bias when reasoning about COVID-19. Journal of experimental psychology. Applied. 2021;27(4):632-656. [31e1371b0f6dc005392bae871c8e52caa11d2feb] | No IHC Key Concepts |
|  | Timmers, Thomas, Janssen, Loes, Pronk, Yvette, van der Zwaard, Babette C, Koeter, Sander, van Oostveen, Dirk, de Boer, Stefan, Kremers, Keetie, Rutten, Sebastiaan, Das, Dirk, van Geenen, Rutger Ci, Koenraadt, Koen Lm, Kusters, Rob, van der Weegen, Walter. Assessing the Efficacy of an Educational Smartphone or Tablet App With Subdivided and Interactive Content to Increase Patients' Medical Knowledge: Randomized Controlled Trial. JMIR mHealth and uHealth. 2018;6(12):e10742. [07a26f873fba4ce50594bf783542dcbdad94d552] | No IHC Key Concepts |
|  | Trost SG, Byrne R, Williams KE, Johnson BJ, Bird A, Simon K, Chai LK, Terranova CO, Christian HE, Golley RK. Study protocol for Healthy Conversations @ Playgroup: a multi-site cluster randomized controlled trial of an intervention to promote healthy lifestyle behaviours in young children attending community playgroups. BMC public health. 2021;21(1):1757. [1f2ac0463ec4b85f374f948ea8c4f4e931c0c42a] | No IHC Key Concepts |
|  | Truman, Emily, Elliott, Charlene. Health-promoting skills for children: Evaluating the influence of a media literacy and food marketing intervention. Health Education Journal. 2020;79(4):431-445. [360eaf3cef43850429b70279d116c21e0b2cd961] | No IHC Key Concepts |
|  | Waters EA, Maki J, Liu Y, Ackermann N, Carter CR, Dart H, Bowen DJ, Cameron LD, Colditz GA. Risk Ladder, Table, or Bulleted List? Identifying Formats That Effectively Communicate Personalized Risk and Risk Reduction Information for Multiple Diseases. Medical decision making : an international journal of the Society for Medical Decision Making. 2021;41(1):74-88. [e77d5afe5251fb54e93ac3ffe7b9f979abe86e3e] | No IHC Key Concepts |
|  | Zinn S, Landrock U, Gnambs T. Web-based and mixed-mode cognitive large-scale assessments in higher education: An evaluation of selection bias, measurement bias, and prediction bias. Behavior research methods. 2021;53(3):1202-1217. [a87c28fddef95c8c408cae04f0c1c4a470ae7cf5] | No IHC Key Concepts |
|  | Aubertin P, Frese T, Kasper J, Mau W, Meyer G, Mikolajczyk R, Richter M, Schildmann J, Steckelberg A. Efficacy of Three Numerical Presentation Formats on Lay People's Comprehension and Risk Perception of Fact Boxes-A Randomized Controlled Pilot Study. International journal of environmental research and public health. 2023;20(3). [d60dc62ef0f09680be3ae55994fd8a715c175150] | No IHC Key Concepts |
|  | Anke Steckelberg. Effectiveness of information presentation in drug fact boxes. 2020; [28851cf73d98e86a55d53859635e2d2e8e8160a7] | No IHC Key Concepts |
|  | van Hoorn, Bastiaan T., van Rossenberg, Luke X., Jacobs, Xander, Sulkers, George S. I., van Heijl, Mark, Ring, David. Clinician Factors Rather Than Patient Factors Affect Discussion of Treatment Options. Clinical Orthopaedics & Related Research®. 2021;479(7):1506-1516. [7cbbdfcd3e8b8acd86c68d36bf97bd27f61acd2a] | No IHC Key Concepts |
|  | Diviani, Nicola, Meppelink, Corine S.. The impact of recommendations and warnings on the quality evaluation of health websites: An online experiment. Computers in Human Behavior. 2017;71:122-129. [e4be88f69c12170e5c4aaa01659636d73633ad4b] | No IHC Key Concepts |
|  | Suwono, H, Permana, T, Saefi, M, Fachrunnisa, R. The problem-based learning (PBL) of biology for promoting health literacy in secondary school students. JOURNAL OF BIOLOGICAL EDUCATION. 2023;57(1):230-244.[4a60bacc022e90672ccc88b926723b41858cd2b8] | No IHC Key Concepts |
|  | Martinez Garcia, L, Alonso-Coello, P, Asso Ministral, L, Balleste-Delpierre, C, Canelo Aybar, C, de Britos, C, Fernandez Rodriguez, A, Gallego Iborra, A, Leo Rosas, V, Llaquet, P, et al.. Learning to make informed health choices: protocol for a pilot study in schools in Barcelona. 2020;8.[bf5d684dd115fb302860be91d0bbbff315e7f356] | Wrong study design |
|  | König, Lars, Marbach-Breitrück, Eugenia, Engler, Anne, Suhr, Ralf. The Development and Evaluation of an e-Learning Course That Promotes Digital Health Literacy in School-age Children: Pre-Post Measurement Study. Journal of Medical Internet Research. 2022;24(5). [4c98780fbba97498306a93b3e5be831ac7563f27] | Wrong study design |
|  | Odierna, Donna H., White, Jenny, Forsyth, Susan, Bero, Lisa A.. Critical appraisal training increases understanding and confidence and enhances the use of evidence in diverse categories of learners. Health Expectations. 2012;18(2):273-287. [4dd36527399559b253122b69f24692b82f1290cf] | Wrong study design |
|  | Alderighi C, Rasoini R, Formoso G, Celani MG, Rosenbaum SE. Feasibility of contextualizing the Informed Health Choices learning resources in Italy: A pilot study in a primary school in Florence. F1000Research. 2022;11:1167.[889146101b3d18d0d4f0692b5c773348949a2925] | Wrong study design |
|  | McGowan BS, Reed JB, Yatcilla JK. Graduate student confidence following a for-credit systematic review course pilot. Journal of the Medical Library Association : JMLA. 2021;109(2):323-329. [2d2f93629834dfbcd0aed7d23caa93b59da5a9f4] | Wrong study design |
|  | Effects of an Inquiry-Oriented Curriculum and Professional Development Program on Grade 7 Students' Understanding of Statistics and on Statistics Instruction. Appendixes. REL 2021-055. Regional Educational Laboratory Southeast. 2021; [29994496ed3367ca2c9d7be1fd26ca71169be400] | Wrong study design |
|  | Nsangi, Allen. Effects of the Informed Health Choices primary school intervention on the ability of children in Uganda to assess the reliability of claims about treatment effects, a cluster-randomised trial. Canadian Conference on Global Health. 2019; [9ad95935bb4c4a08568118b350245c24e344ced8] | Wrong study design |
|  | Jimenez-Chavez, JC, Rosario-Maldonado, FJ, Torres, JA, Ramos-Lucca, A, Castro-Figueroa, EM, Santiago, L. Assessing Acceptability, Feasibility, and Preliminary Effectiveness of a Community-Based Participatory Research Curriculum for Community Members: A Contribution to the Development of a Community-Academia Research Partnership. HEALTH EQUITY. 2018;2(1):272-281 [edd0c522546383bdf0c4699546a1106b714684b5] | Wrong study design |
|  | Nash, R, Elmer, S, Thomas, K, Osborne, R, MacIntyre, K, Shelley, B, Murray, L, Harpur, S, Webb, D. HealthLit4Kids study protocol; crossing boundaries for positive health literacy outcomes. BMC PUBLIC HEALTH. 2018;18 [1b8c64321344fecbf714b64ecdd26b550a039a43] | Wrong study design |
|  | Camilla Alderighi, Raffaele Rasoini, Giulio Formoso, Maria Grazia Celani, & Sarah E. Rosenbaum.. Feasibility of contextualizing the Informed Health Choices learning resources in Italy: A pilot study in a primary school in Florence. 2022; [66db0f638cf176fe5e9fac3688736462232024e2] | Wrong study design |
|  | Lapointe, L. Impact of a course in evidence-informed policy-making on the acquisition of methodological knowledge: Findings from before-and-after studies conducted on three consecutive cohorts of master students. TEACHING PUBLIC ADMINISTRATION. 2019;37(3):293-311.[3d3e73fd90650379230ff9485e0986d0b405f5b1] | Wrong study design |
|  | Han, GN, Mayer, M, Canner, J, Lindsley, K, Datar, R, Le, J, Bar-Cohen, A, Bowie, J, Dickersin, K. Development, implementation and evaluation of an online course on evidence-based healthcare for consumers. BMC HEALTH SERVICES RESEARCH. 2020;20(1). [dd14dd5570aa0496955e86299571cddee8e11551] | Wrong study design |
|  | Does the use of the Informed Health Choices teaching resources improve the secondary students’ ability to critically think about health in Uganda? A cluster randomized trial protocol. Pan African Clinical Trials Registry. 2022; [[40833862677380f968fb6e0614f3c3f38a9af90a](https://www.epistemonikos.org/en/documents/40833862677380f968fb6e0614f3c3f38a9af90a)] | Trial registry with no results reported |
|  | Informed Health Choices. Pan African Clinical Trials Registry. 2022; [[cf9866052639d82fd3c5d127ff3ce3a054fc41d6](https://www.epistemonikos.org/en/documents/cf9866052639d82fd3c5d127ff3ce3a054fc41d6)] | Trial registry with no results reported |
|  | Study to measure the impact of a speaking book on patient Understanding of clinical research knowledge. Clinical Trials Registry - India. 2024; [[96c03599793dc205f3d23d8fad93d13c4a1e10e7](https://www.epistemonikos.org/en/documents/96c03599793dc205f3d23d8fad93d13c4a1e10e7)] | Trial registry with no results reported |
|  | Testing the effect of digital secondary school resources for informed health choices among secondary school students in Rwanda. Pan African Clinical Trials Registry. 2022; [[0f14ee604dbd2790040a2eea1c2276f747e398f0](https://www.epistemonikos.org/en/documents/0f14ee604dbd2790040a2eea1c2276f747e398f0)] | Trial registry with no results reported |
|  | lifestyle education based on health thinking to preschool children and parents. Iranian Registry of Clinical Trials. 2023; [[b7f90c065afbd7cc33b127586b84b6850ce01a87](https://www.epistemonikos.org/en/documents/b7f90c065afbd7cc33b127586b84b6850ce01a87)] | Trial registry with no results reported |
|  | Abdoh E. Online health information seeking and digital health literacy among information and learning resources undergraduate students. Journal of academic librarianship. 2022;48(6):102603. [[32f6b064e497c028673d84860ec2b449d5c8768e](https://www.epistemonikos.org/en/documents/32f6b064e497c028673d84860ec2b449d5c8768e)] | There was no assessment of the effect of educational interventions aimed at improving people’s understanding of KCCHA. |
|  | Alhuthail A, Aloraini B, Alhuthail I, Alrashidi SM, Alotibi RM. Attitudes and Awareness Regarding Health Information Sought on Social Media by the Saudi Population: A Cross-Sectional Study. Cureus. 2025;17(1):e77759. [[2622869ea3f68622ab4f59ebcff452c170613851](https://www.epistemonikos.org/en/documents/2622869ea3f68622ab4f59ebcff452c170613851)] | Not the study design |
|  | Alnuaim A. The Impact and Acceptance of Gamification by Learners in a Digital Literacy Course at the Undergraduate Level: Randomized Controlled Trial. JMIR serious games. 2024;12:e52017. [[51958807c67b56e88d340bb6dec12f54fe9d79ff](https://www.epistemonikos.org/en/documents/51958807c67b56e88d340bb6dec12f54fe9d79ff)] | There was no assessment of the effect of educational interventions aimed at improving people’s understanding of KCCHA. |
|  | Amir Rastpour, Abraham Amini. Age Guessing: A Game to Introduce Fundamental Statistical Concepts. Journal of Statistics and Data Science Education. 2025;33(1):62-67. [[92f7fa3a782cf224d79b508317dc1640b501d285](https://www.epistemonikos.org/en/documents/92f7fa3a782cf224d79b508317dc1640b501d285)] | There was no assessment of the effect of educational interventions aimed at improving people’s understanding of KCCHA. |
|  | Angelelli CV, Ribeiro GMC, Severino MR, Johnstone E, Borzenkova G, da Silva DCO. Developing critical thinking skills through gamification. Thinking skills and creativity. 2023;49:101354. [[41a5f8a4c81c1606ee42162e907ba0360ef9d289](https://www.epistemonikos.org/en/documents/41a5f8a4c81c1606ee42162e907ba0360ef9d289)] | There was no assessment of the effect of educational interventions aimed at improving people’s understanding of KCCHA. |
|  | Anna Khalemsky, Roy Gelbard, Yelena Stukalin. Constructing a Course on Classification Methods for Undergraduate Non-Stem Students: Striving to Reach Knowledge Discovery. Journal of Statistics and Data Science Education. 2025;33(1):68-76. [[9cc56a258f26d6b49c06968867487f3d34aa0371](https://www.epistemonikos.org/en/documents/9cc56a258f26d6b49c06968867487f3d34aa0371)] | There was no assessment of the effect of educational interventions aimed at improving people’s understanding of KCCHA. |
|  | Ayre J, Bonner C, Muscat DM, Cvejic E, Mac O, Mouwad D, Shepherd HL, Aslani P, Dunn AG, McCaffery KJ. Online Plain Language Tool and Health Information Quality: A Randomized Clinical Trial. JAMA network open. 2024;7(10):e2437955. [[3dbedec62724a3cfc1c3699e04923e446b765134](https://www.epistemonikos.org/en/documents/3dbedec62724a3cfc1c3699e04923e446b765134)] | There was no assessment of the effect of educational interventions aimed at improving people’s understanding of KCCHA. |
|  | Ayre, J, Bonner, C, Muscat, DM, Cvejic, E, Mac, O, Mouwad, D, Shepherd, HL, Aslani, P, Dunn, AG, McCaffery, KJ. Online Plain Language Tool and Health Information Quality. JAMA NETWORK OPEN. 2024;7(10). [[0ea88d72323e30e902ed4e85b6eaef37656d4cc5](https://www.epistemonikos.org/en/documents/0ea88d72323e30e902ed4e85b6eaef37656d4cc5)] | There was no assessment of the effect of educational interventions aimed at improving people’s understanding of KCCHA. |
|  | Domu, Ichdar, Pinontoan, Kinzie Feliciano, Mangelep, Navel Oktaviandy. Problem-Based Learning in the Online Flipped Classroom: Its Impact on Statistical Literacy Skills. Journal of Education and e-Learning Research. 2023;10(2):336-343. [[f6a24f34c089194f22994fd8564830d67d00f6fc](https://www.epistemonikos.org/en/documents/f6a24f34c089194f22994fd8564830d67d00f6fc)] | There was no assessment of the effect of educational interventions aimed at improving people’s understanding of KCCHA. |
|  | Dwyer, Christopher P. An Evaluative Review of Barriers to Critical Thinking in Educational and Real-World Settings. Journal of Intelligence. 2023;11(6). [[04c6aa4d8cdd6e2c6d4966d15ea52245765ca94e](https://www.epistemonikos.org/en/documents/04c6aa4d8cdd6e2c6d4966d15ea52245765ca94e)] | There was no assessment of the effect of educational interventions aimed at improving people’s understanding of KCCHA. |
|  | Fleary SA, Joseph PL, Rastogi S, Fenton T, Srivastava V. A Study on How Libraries Operate as Health Spaces in the United States. Journal of community health. 2024; [[602bbdfd3a4d986fa275633e070d51c03b8fab0a](https://www.epistemonikos.org/en/documents/602bbdfd3a4d986fa275633e070d51c03b8fab0a)] | There was no assessment of the effect of educational interventions aimed at improving people’s understanding of KCCHA. |
|  | Garg, Vandana, Alcasid, Zee, Mendoza, Katherine, Lee, Heesoo, Loo, Yi Xin, Nong, Andy, Toh, Gerard W, Tan, Sheryl. Use of the Consumer Health Literacy Quotient to Quantify and Explore Self-Care Readiness Among Consumers in Four Asia-Pacific Countries. Healthcare (Basel, Switzerland). 2024;12(22). [[9101ee37b3b7d23ab94b2cb645eed56386892b46](https://www.epistemonikos.org/en/documents/9101ee37b3b7d23ab94b2cb645eed56386892b46)] | There was no assessment of the effect of educational interventions aimed at improving people’s understanding of KCCHA. |
|  | Gentizon, Jenny, Fleury, Mapi, Pilet, Eric, Bula, Christophe, Mabire, Cedric. Conceptualization and content validation of the MEDication literacy assessment of geriatric patients and informal caregivers (MED-fLAG). Journal of patient-reported outcomes. 2022;6(1):87. [[38ccd5725f102bbb6f8e1831880f64a8c2c89d78](https://www.epistemonikos.org/en/documents/38ccd5725f102bbb6f8e1831880f64a8c2c89d78)] | There was no assessment of the effect of educational interventions aimed at improving people’s understanding of KCCHA. |
|  | Holzmann, Ursula, Anand, Sulekha, Payumo, Alexander Y. The ChatGPT Fact-Check: Exploiting the Limitations of Generative AI to Develop Evidenced-Based Reasoning Skills in College Science Courses. Advances in physiology education. 2025; [[fb0bb20f6651d70752539fa3988471358369bf52](https://www.epistemonikos.org/en/documents/fb0bb20f6651d70752539fa3988471358369bf52)] | There was no assessment of the effect of educational interventions aimed at improving people’s understanding of KCCHA. |
|  | Hsu, Hua-I, Liu, Chih-Chi, Yang, Stephanie Fu, Chen, Hsueh-Chih. A health promotion program for older adults (KABAN!): effects on health literacy, quality of life, and emotions. Educational Gerontology. 2023;49(8):639-656. [[28984aabcc794a9e410b6349c5f04c0c48fbe60f](https://www.epistemonikos.org/en/documents/28984aabcc794a9e410b6349c5f04c0c48fbe60f)] | There was no assessment of the effect of educational interventions aimed at improving people’s understanding of KCCHA. |
|  | Huang L, Jin Y, Chien CW, Xu Q, Chuang YC, Tung TH. Exploring the Health Literacy Behavior Patterns of Male Patients Using an Interpretable Method. Risk management and healthcare policy. 2024;17:2099-2109. [[39bc96efb4b796e74c092dfa53c56403134919c2](https://www.epistemonikos.org/en/documents/39bc96efb4b796e74c092dfa53c56403134919c2)] | There was no assessment of the effect of educational interventions aimed at improving people’s understanding of KCCHA. |
|  | Isautier, Jennifer, Webster, Angela C, Lambert, Kelly, Shepherd, Heather L, McCaffery, Kirsten, Sud, Kamal, Kim, Jinman, Liu, Na, De La Mata, Nicole, Raihana, Shahreen, Kelly, Patrick J, Muscat, Danielle M, SUCCESS Study Group, Foote C, Huang R, Kanagaratnam R, Mac K, Mai J, O'Lone E, Robbins A, Saunders J, Zwi S. Evaluation of the SUCCESS Health Literacy App for Australian Adults With Chronic Kidney Disease: Protocol for a Pragmatic Randomized Controlled Trial. JMIR research protocols. 2022;11(8):e39909. [[db086884992335c6ecd168852e066ad8b887fc9e](https://www.epistemonikos.org/en/documents/db086884992335c6ecd168852e066ad8b887fc9e)] | There was no assessment of the effect of educational interventions aimed at improving people’s understanding of KCCHA. |
|  | Jennifer Hill, George Perrett, Stacey A. Hancock, Le Win, Yoav Bergner. Causal Language and Statistics Instruction: Evidence from a Randomized Experiment. Statistics Education Research Journal. 2024;23(1). [[5e4c20d9b08051103e631da25e4367b35f233289](https://www.epistemonikos.org/en/documents/5e4c20d9b08051103e631da25e4367b35f233289)] | There was no assessment of the effect of educational interventions aimed at improving people’s understanding of KCCHA. |
|  | Julianne Foxworthy Gonzalez. "I No Longer Skip the Numbers." An Analysis of Students' Use of Statistical Literacy Practices. ProQuest LLC. 2024; [[19c483836a63757b6be688e5f601f105f354d656](https://www.epistemonikos.org/en/documents/19c483836a63757b6be688e5f601f105f354d656)] | Not the study design |
|  | Krzyz, Ewa Zuzanna, Putri, Anggie Pradana, Lin, Hung-Ru. Factors Influencing Health Literacy Among Migrants in Taiwan. Journal of transcultural nursing : official journal of the Transcultural Nursing Society. 2024;:10436596241301397. [[b3b6f221f8ce1d2ed74550318908625753299614](https://www.epistemonikos.org/en/documents/b3b6f221f8ce1d2ed74550318908625753299614)] | There was no assessment of the effect of educational interventions aimed at improving people’s understanding of KCCHA. |
|  | Laura Rosof, Julie Edmunds, Dora Gicheva. Impact of STEM Mentored Undergraduate Research Program at a Community College: Results from an RCT. Society for Research on Educational Effectiveness. 2024; [[1cda8135627f3bdd15ff04139834a870b956144f](https://www.epistemonikos.org/en/documents/1cda8135627f3bdd15ff04139834a870b956144f)] | There was no assessment of the effect of educational interventions aimed at improving people’s understanding of KCCHA. |
|  | Levy DA, Jordan HS, Lalor JP, Smirnova JK, Hu W, Liu W, Yu H. Individual Factors That Affect Laypeople's Understanding of Definitions of Medical Jargon. Health policy and technology. 2024;13(6). [[f46cc25310dc677bb6a0d253dca4e146b8d3b0e6](https://www.epistemonikos.org/en/documents/f46cc25310dc677bb6a0d253dca4e146b8d3b0e6)] | There was no assessment of the effect of educational interventions aimed at improving people’s understanding of KCCHA. |
|  | Li, Mengqi, Devane, Declan, Beecher, Claire, Duffy, Austin G, Duggan, Caitriona, Dowling, Maura, Grimes, David Robert, Kennan, Avril, McLoughlin, Sarah, Nsangi, Allen, Oxman, Andrew D, O'Connor, Robert, Stewart, Derek C, Toomey, Elaine, Tierney, Marie. Prioritising Informed Health Choices Key Concepts for those impacted by cancer: a protocol. HRB open research. 2022;5:55. [[457c6ddc66ea4c4cd704b01faa26f7dbb2193d14](https://www.epistemonikos.org/en/documents/457c6ddc66ea4c4cd704b01faa26f7dbb2193d14)] | Protocol. |
|  | Lies Sercu. Measuring HL in Adolescent Students: A Preliminary Report on the Development of a Task- and Skills-Based Assessment Instrument for Use in Educational Settings. Discover Education. 2023;2(1). [[26ab23e7eb3199dbbb5cc98770043e1ff12181be](https://www.epistemonikos.org/en/documents/26ab23e7eb3199dbbb5cc98770043e1ff12181be)] | There was no assessment of the effect of educational interventions aimed at improving people’s understanding of KCCHA. |
|  | London School of Hygiene and Tropical Medicine. Every Newborn-Reach Up Early Education Intervention for All Children- a Parent Group Intervention for School Readiness in Bangladesh, Nepal, and Tanzania. clinicaltrials.gov. 2022; [[77cd0df93a74208b519c63075807c493cb11ad67](https://www.epistemonikos.org/en/documents/77cd0df93a74208b519c63075807c493cb11ad67)] | Trial registry with no results reported |
|  | MacDonald H, Bezaire V. Leveraging Systematic Review Practice for Research Skill Development in an Undergraduate Science Course: A Case Study. Advances in physiology education. 2024;48(3):518-526. [[71858267137bd181b79dbf0b3b0ffa72064f50da](https://www.epistemonikos.org/en/documents/71858267137bd181b79dbf0b3b0ffa72064f50da)] | Not the study design |
|  | Mendoza H, D'Agostino McGowan L. Randomized controlled trial: Quantifying the impact of disclosing uncertainty on adherence to hypothetical health recommendations. PloS one. 2022;17(12):e0278263. [[41afcceb8301accd688d46f4f374a5ff283159f0](https://www.epistemonikos.org/en/documents/41afcceb8301accd688d46f4f374a5ff283159f0)] | There was no assessment of the effect of educational interventions aimed at improving people’s understanding of KCCHA. |
|  | Nazari A, Askari A, Rahimi Foroushani A, Garmaroudi G. The effect of educational intervention based on social media on mental health literacy of high school students in Ramhormoz city: study protocol of a randomized controlled trial. Frontiers in psychology. 2024;15:1377760. [[8e0c83ad4637888ec545410c25b402590b47ab7a](https://www.epistemonikos.org/en/documents/8e0c83ad4637888ec545410c25b402590b47ab7a)] | Protocol |
|  | Oniani D, Sreekumar S, DeAlmeida R, DeAlmeida D, Hui V, Lee YJ, Zhang Y, Zhou L, Wang Y. Toward Improving Health Literacy in Patient Education Materials with Neural Machine Translation Models. AMIA Joint Summits on Translational Science proceedings. AMIA Joint Summits on Translational Science. 2023;2023:418-426. [[3f1fe7be99bcc45e271ed9e29b7e63932ffb8a5f](https://www.epistemonikos.org/en/documents/3f1fe7be99bcc45e271ed9e29b7e63932ffb8a5f)] | There was no assessment of the effect of educational interventions aimed at improving people’s understanding of KCCHA. |
|  | Ooi C, Nalliah S. Harnessing Reliable Evidence in the Post-COVID Era: A Practice Guide to Navigating the Ocean of Medical Literature. Cureus. 2024;16(1):e52746. [[e0b53d51b75e1c620c42a37de41ae176fb3ae5ed](https://www.epistemonikos.org/en/documents/e0b53d51b75e1c620c42a37de41ae176fb3ae5ed)] | There was no assessment of the effect of educational interventions aimed at improving people’s understanding of KCCHA. |
|  | Parker AE, Scull TM, Kennedy KL. Efficacy of DigiKnowItNews: Teen, a multimedia educational website for adolescents about pediatric clinical trials: study protocol for a randomized controlled trial. Trials. 2023;24(1):436. [[229a225de7a5a7962be4e2ec4d40baf9cfea61d5](https://www.epistemonikos.org/en/documents/229a225de7a5a7962be4e2ec4d40baf9cfea61d5)] | Protocol |
|  | Poudyal BS, Dulal S, Shilpakar R, Gyawali B. Highlights from ecancer Choosing Wisely Nepal 2022: critical appraisal skills for evidence-based practice, 24th-25th September 2022, Kathmandu, Nepal. Ecancermedicalscience. 2022;16:1478. [[fe6a8aa0dabab4d4fc3574e0d098e50896a3c009](https://www.epistemonikos.org/en/documents/fe6a8aa0dabab4d4fc3574e0d098e50896a3c009)] | There was no assessment of the effect of educational interventions aimed at improving people’s understanding of KCCHA. |
|  | Rachmani E, Haikal H, Rimawati E. Development and validation of digital health literacy competencies for citizens (DHLC), an instrument for measuring digital health literacy in the community. Computer methods and programs in biomedicine update. 2022;2:100082. [[debbeea2712c1417ee3d5025f1274fcaa0c32d49](https://www.epistemonikos.org/en/documents/debbeea2712c1417ee3d5025f1274fcaa0c32d49)] | There was no assessment of the effect of educational interventions aimed at improving people’s understanding of KCCHA. |
|  | Reddy, Anireddy R, Doshi, Anushree K, Mak, Allison, Shea, Judy A, Fardad, Joana T, Moon, Jiwon, Hu, Paula, Garcia-Marcinkiewicz, Annery G. Assessing the health literacy of caregivers in the pediatric intensive care unit: a mixed-methods study. Frontiers in pediatrics. 2023;11:1308673. [[89ee46cb2eac8aefdd1b59f59af8bbe6c103bed9](https://www.epistemonikos.org/en/documents/89ee46cb2eac8aefdd1b59f59af8bbe6c103bed9)] | There was no assessment of the effect of educational interventions aimed at improving people’s understanding of KCCHA. |
|  | Steel, Amie, Foley, Hope, Bugarcic, Andrea, Wardle, Jon, Boyd, Hannah, Breakspear, Ian, Carlton, Anne-Louise, Cope, Greg, Dua, Kamal, Greenway, Patricia, Redmond, Rebecca, Hawrelak, Jason, Shukla, Naveen, Adams, Jon. Exploring Criteria for the Translation of Traditional Knowledge Within Contemporary Clinical Practice, Research, Policy, and Education: A Stakeholder Forum. Journal of integrative and complementary medicine. 2023;29(6-7):348-360. [[1632a42cdb5125c6a10ddfdab81922dde3e72386](https://www.epistemonikos.org/en/documents/1632a42cdb5125c6a10ddfdab81922dde3e72386)] | There was no assessment of the effect of educational interventions aimed at improving people’s understanding of KCCHA. |
|  | Sy-Miin Chow, Jungmin Lee, Jonathan Park, Prabhani Kuruppumullage Don, Tracey Hammel, Michael N. Hallquist, Eric A. Nord, Zita Oravecz, Heather L. Perry, Lawrence M. Lesser, Dennis K. Pearl. Personalized Education through Individualized Pathways and Resources to Adaptive Control Theory-Inspired Scientific Education (iPRACTISE): Proof-of-Concept Studies for Designing and Evaluating Personalized Education. Journal of Statistics and Data Science Education. 2024;32(2):174-187. [[4b37a3dbff63c8c935fa64e5cc45fd4bdc1712eb](https://www.epistemonikos.org/en/documents/4b37a3dbff63c8c935fa64e5cc45fd4bdc1712eb)] | There was no assessment of the effect of educational interventions aimed at improving people’s understanding of KCCHA. |
|  | Sydney Health Literacy Lab at the University of Sydney. The Effects of a Short Video Intervention on Critically Appraising Online Health Information. ANZCTR. 2023; [[a1235f47e64dd88567cb20afbbc9b4006832beb6](https://www.epistemonikos.org/en/documents/a1235f47e64dd88567cb20afbbc9b4006832beb6)] | Trial registry with no results reported |
|  | Tara Boelsen-Robinson. Testing tools to support the A Better Choice Strategy Implementation: the CREATE study. ANZCTR. 2023; [[f53f166dd1be1d238a35afa2e16793c0414394ae](https://www.epistemonikos.org/en/documents/f53f166dd1be1d238a35afa2e16793c0414394ae)] | Trial registry with no results reported |
|  | Victoria L. Cross, Megan N. Imundo, Courtney M. Clark, Melissa Paquette-Smith. Is There a Main Effect? Improving Data Literacy Using Practice Examples and Peer Collaboration. Psychology Learning and Teaching. 2024;23(2):172-188. [[f7575f008ec2cfcf04168540fa6ed57c1e455dd3](https://www.epistemonikos.org/en/documents/f7575f008ec2cfcf04168540fa6ed57c1e455dd3)] | There was no assessment of the effect of educational interventions aimed at improving people’s understanding of KCCHA. |
|  | Wu, Shuqiang, Shao, Bilin, Wang, Gaimei. Health Literacy Among University Students in Shaanxi Province of China: A Cross-Sectional Study. Risk management and healthcare policy. 2023;16:865-878. [[d60aee3fee88fcd648f3d112c65345bed2dcc921](https://www.epistemonikos.org/en/documents/d60aee3fee88fcd648f3d112c65345bed2dcc921)] | Not the study design |
|  | Xie W, Xiao J, Chen J, Huang H, Huang X, He S, Xu L. Impact of health education on promoting influenza vaccination health literacy in primary school students: a cluster randomised controlled trial protocol. BMJ open. 2024;14(4):e080115. [[10e106b0f8ae71e4d841ceda84727400e5875c93](https://www.epistemonikos.org/en/documents/10e106b0f8ae71e4d841ceda84727400e5875c93)] | There was no assessment of the effect of educational interventions aimed at improving people’s understanding of KCCHA. |
|  | Zhang, Xiaodong, Liu, Li, You, Xiaowei, Wang, Yiran. The impact of blended teaching based on learning communication on students' academic performance and critical thinking ability. Minerva medica. 2024; [[ae5acf19249dc1cc0c7a3ca52ff169df5ab41d91](https://www.epistemonikos.org/en/documents/ae5acf19249dc1cc0c7a3ca52ff169df5ab41d91)] | There was no assessment of the effect of educational interventions aimed at improving people’s understanding of KCCHA. |
|  | Zorgo, Szilvia, Peters, Gjalt-Jorn, Jeney, Anna, Shaffer, David Williamson, Ruis, Andrew R, Crutzen, Rik. A feasibility study for a unified, multimodal analysis of online information foraging in health-related topics. Open research Europe. 2023;3:98. [[96e23445431f8e9c1e815529fef34796a4df4f15](https://www.epistemonikos.org/en/documents/96e23445431f8e9c1e815529fef34796a4df4f15)] | There was no assessment of the effect of educational interventions aimed at improving people’s understanding of KCCHA. |
|  | Kambara K, Matsumoto M, Hako S, Shigematsu J, Yokoyama S, Ogata A. An intervention to promote concrete thinking style in young adults: effects on depressive symptoms and its protective factors. Journal of behavior therapy and experimental psychiatry. 2023;81:101857. [[3660ab764066d0a9662a0a917c8c79a12e581947](https://www.epistemonikos.org/en/documents/3660ab764066d0a9662a0a917c8c79a12e581947)] | There was no assessment of the effect of educational interventions aimed at improving people’s understanding of KCCHA. |
|  | Lyons B, King AJ, Kaphingst KA. A Health Media Literacy Intervention Increases Skepticism of Both Inaccurate and Accurate Cancer News Among U.S. Adults. Annals of behavioral medicine : a publication of the Society of Behavioral Medicine. 2024;58(12):820‐831. [[a70def95b4dbbb26765732e9aaafbd5e16623c3d](https://www.epistemonikos.org/en/documents/a70def95b4dbbb26765732e9aaafbd5e16623c3d)] | There was no assessment of the effect of educational interventions aimed at improving people’s understanding of KCCHA. |
|  | Lühnen J, Haastert B, Richter T. Informed Decision-Making with and for People with Dementia-Efficacy of the PRODECIDE Education Program for Legal Representatives: A Randomized Controlled Trial (PRODECIDE-RCT) and Process Evaluation. Geriatrics (Basel, Switzerland). 2024;9(3). [[8c04dea818dbbae99ee51170b73f69f1b22c5f58](https://www.epistemonikos.org/en/documents/8c04dea818dbbae99ee51170b73f69f1b22c5f58)] | There was no assessment of the effect of educational interventions aimed at improving people’s understanding of KCCHA. |
|  | Orosz, G, Faragó, L, Paskuj, B, Krekó, P. Strategies to combat misinformation: Enduring effects of a 15-minute online intervention on critical-thinking adolescents. Computers in human behavior. 2024;159:N.PAG-N.PAG. [[57a3174df5269844ff661726a3691232db1f830a](https://www.epistemonikos.org/en/documents/57a3174df5269844ff661726a3691232db1f830a)] | There was no assessment of the effect of educational interventions aimed at improving people’s understanding of KCCHA. |
|  | Srisaknok, T, Ploylearmsang, C, Wongkongdech, R. Effectiveness of Program for Older People's Health Literacy on Drug and Health Products: Northeast of Thailand. ASIAN JOURNAL OF SOCIAL HEALTH AND BEHAVIOR. 2024;7(1):11-18. [[7d72c026ba751432983f63f4a06bc676d32a6d2d](https://www.epistemonikos.org/en/documents/7d72c026ba751432983f63f4a06bc676d32a6d2d)] | There was no assessment of the effect of educational interventions aimed at improving people’s understanding of KCCHA. |
|  | Chesire F, Mugisha M, Ssenyonga R, Rose CJ, Nsangi A, Kaseje M, Sewankambo NK, Oxman M, Rosenbaum SE, Moberg J, Dahlgren A, Lewin S, Oxman AD. Effects of the Informed Health Choices secondary school intervention: a prospective meta-analysis. Journal of evidence-based medicine. 2023;16(3):321-331. [7241399f0f5e0d17d0ffd784091946259171c0e1] | Not the study design |
|  | Chesire F, Mugisha M, Ssenyonga R, Rose CJ, Nsangi A, Kaseje M, Sewankambo NK, Oxman M, Rosenbaum SE, Moberg J, Dahlgren A, Lewin S, Venkateswaran M, Papadopoulou E, Oxman AD. Effects of the informed health choices secondary school intervention after 1 year: a prospective meta-analysis using individual participant data. Trials. 2024;25(1):733. [[0cf51f65689be02f0dec80000432357a580c1722](https://www.epistemonikos.org/en/documents/0cf51f65689be02f0dec80000432357a580c1722)] | Not the study design |
|  | Yamazaki, KG, Taylor, A, Asikin-Garmager, A, Han, SR, Bartlett, L. Use of All of Us data to increase health literacy and research skills in high school students. JOURNAL OF THE AMERICAN MEDICAL INFORMATICS ASSOCIATION. 2024;31(12):3001-3007. [[3ee023d18bba9838d82c0f5eb88eac82696b67c8](https://www.epistemonikos.org/en/documents/3ee023d18bba9838d82c0f5eb88eac82696b67c8) | There was no assessment of the effect of educational interventions aimed at improving people’s understanding of KCCHA. |
|  | Elvsaas IO, Garnweidner-Holme L, Habib L, Molin M. Development and Evaluation of a Serious Game Application to Engage University Students in Critical Thinking About Health Claims: Mixed Methods Study. JMIR formative research. 2023;7:e44831. [[d03096f802db7d8ae5153dff3b4b38f76d660b71](https://www.epistemonikos.org/en/documents/d03096f802db7d8ae5153dff3b4b38f76d660b71)] | There was no assessment of the effect of educational interventions aimed at improving people’s understanding of KCCHA. |
|  | Li, Mengqi, Devane, Declan, Beecher, Claire, Dowling, Maura, Duffy, Austin G, Duggan, Caitriona, Grimes, David Robert, Kennan, Avril, Kilty, Claire, Nsangi, Allen, Oxman, Andrew D, Stewart, Derek C, Toomey, Elaine, Tierney, Marie. Prioritising Key Concepts for informed health choices in cancer: An evidence-based online educational programme. PEC innovation. 2024;5:100311. [[3d630fe31f2d751d8593f602a5266d3463f8bc2c](https://www.epistemonikos.org/en/documents/3d630fe31f2d751d8593f602a5266d3463f8bc2c)] | There was no assessment of the effect of educational interventions aimed at improving people’s understanding of KCCHA. |
|  | List, John A, Ramirez, Lina M, Seither, Julia, Unda, Jaime, Vallejo, Beatriz H. Critical thinking and misinformation vulnerability: experimental evidence from Colombia. PNAS nexus. 2024;3(10):pgae361. [[1f6e316294faeaa12b815688d203dea3d2928156](https://www.epistemonikos.org/en/documents/1f6e316294faeaa12b815688d203dea3d2928156)] | There was no assessment of the effect of educational interventions aimed at improving people’s understanding of KCCHA. |
|  | Londra, Franco, Saux, Gaston. The Effect of Document Source Trustworthiness on the Evaluation and Strategic Use of Embedded Sources When Reading Health Information Online. Reading Psychology. 2023;44(6):623-648. [[f9fe6653a72c6c0d0ad809182a25327cee35a38c](https://www.epistemonikos.org/en/documents/f9fe6653a72c6c0d0ad809182a25327cee35a38c)] | There was no assessment of the effect of educational interventions aimed at improving people’s understanding of KCCHA. |
|  | Shah AR, Ni L, Bay AA, Hart AR, Perkins MM, Hackney ME. Remote versus In-Person Health Education: Feasibility, Satisfaction, and Health Literacy for Diverse Older Adults. Health education & behavior : the official publication of the Society for Public Health Education. 2023;50(3):10901981221121258. [[d4731cf54dbdc93bf8a043e8885fd5356dbc7621](https://www.epistemonikos.org/en/documents/d4731cf54dbdc93bf8a043e8885fd5356dbc7621)] | There was no assessment of the effect of educational interventions aimed at improving people’s understanding of KCCHA. |

IHC = Informed Health Choices
