# Supplementary material for: Do studies of interventions to improve laypeople’s critical thinking about health choices assess potential harms? A systematic review
Source: BMJ Open. 2026 Apr 24;16(4):e108268. doi: 10.1136/bmjopen-2025-108268 (PMC13110562; doi:10.1136/bmjopen-2025-108268)
Supplement: Supplementary file 5 [file bmjopen-2025-108268.reviewer_comments.pdf]

PEER REVIEW HISTORY

BMJ Open publishes all reviews undertaken for accepted manuscripts. Reviewers are asked to complete a checklist review form and are provided with free text boxes to elaborate on their assessment. These free text comments are reproduced below.

ARTICLE DETAILS

Title (Provisional)

DO STUDIES OF INTERVENTIONS TO IMPROVE LAYPEOPLE'S CRITICAL THINKING ABOUT HEALTH CHOICES ASSESS POTENTIAL HARMS? A SYSTEMATIC REVIEW.

Authors

Oxman, Matt; Cusack, Leila; Verdugo-Paiva, Francisca; Ávila, Camila; Peña, Javiera; Novillo, Francisco; Oxman, Andrew D; Fretheim, Atle; Melby-Lervåg, Monica; Nordheim, Lena

---

VERSION 1 - REVIEW

**Reviewer**	**1**
**Name**	**Vogt, Randi**
**Affiliation**	**Geisinger, Bioethics and Decisions Sciences**
**Date**	**21-Aug-2025**
**COI**	**None**

Thank you for the opportunity to review the manuscript titled “Do studies of interventions to improve laypeople’s critical thinking about health choices assess potential harms? A systematic review” (bmjopen-2025-108268). The overall research question investigated is how often researchers report adverse effects of educational interventions intended to improve people’s abilities to think critically about health choices. The authors find that only one out of the 35 papers they identified reported adverse effects. This was an interesting paper that could have some implications for how readers should evaluate these interventions as well as some important recommendations for how researchers should report adverse events associated with the interventions. The following are some questions and suggestions that the authors might consider in their revision.

Abstract

In the abstract, the authors say that this study “was an expansion of an earlier systematic review” and then say it is the “first systematic review focusing on adverse effects of any category of educational interventions.” This is confusing. Is this study the first review of its kind or does it build upon a previous review? In general (in the abstract as well as in the

introduction and discussion), the novelty of this study above and beyond what has been done previously should be more clearly highlighted.

Introduction

The introduction includes an example of a health intervention an educational intervention for reading with adverse effects but an example of an educational intervention for a health choice (and an adverse effect of that intervention) would be more useful.

I found Table 1 to be quite confusing. Are 1.1, 2.1, and 3.1 supposed to relate to each other since they're in the same row? Changing the orientation so that the row headers are column headers might help.

Methods

The authors should address how the non-inclusion of unpublished studies might affect the results. It could be that those papers do assess and report adverse effects but that makes the paper/intervention seem weaker so it hasn't been published.

The authors should clearly define what an adverse effect is in this context. On p. 6, they say "we extracted any mention of potential adverse effects" but it is not clear how they decided whether something was an adverse effect. In other words, what instructions were coders given on how to identify a mention of an adverse effect? Did the phrase adverse effect specifically need to be mentioned or could an adverse effect be inferred from context clues?

Results

It would be useful to add a column to Table 4 showing which of the "Key concepts for informed health choices" that study addressed.

In Table 5, are the results presented the results of the adverse effect? Meaning, there was no differences between intervention groups on their experience of the potential adverse effect? This should be made more clear.

The authors might consider bolding the text in Table 6 that relates to adverse effects as some of the quotes are long and some of the text is introductory rather than substantive.

At the top of p. 13, the authors report that the number of studies as well as the percentage (1/35, 3%) that meet the criteria for that analysis. For consistency, the authors might consider adding a percentage to the result in the middle of p. 13 and at the top of p. 16.

Based on Table 7, it seems that many protocols mention the possibility of adverse effects but then those effects (even the absence of them) are not reported in the published studies. The authors should discuss this disconnect between the protocol and the published study. Is it possible that adverse effects were not mentioned because there were no adverse effects in that study? Do the authors recommend that all studies include a statement about adverse effects, even just to acknowledge that there were none?

Discussion

The ”Other evidence” section feels out of place. Most of the evidence in this section does not relate to health choices/interventions. If the authors wish to keep this section, they need to make it more clear how this relates to the findings they present earlier.

In the “Strengths” section, the authors conclude “the results clearly suggest researchers generally have not considered or assessed potential adverse effects…” However, Table 7 suggests that most researchers have considered potential adverse effects but have not reported them in their published study.

Supplementary Files

The table(s) presented in the Supplementary Files starting on page 52 of 131 of the PDF are very difficult to piece together across multiple pages.

**Reviewer**	**2**
**Name**	**Sornlorm, Kittipong**
**Affiliation**	**Khon Kaen University, Faculty of Public Health, Khon Kaen University**
**Date**	**28-Nov-2025**
**COI**	**None**

This manuscript addresses a significant and often overlooked aspect of educational interventions: the potential for unintended harms. By systematically reviewing studies on critical thinking interventions to assess whether adverse effects are measured, the authors identify a critical gap in the current evidence base. The finding that 94% of studies failed to assess potential harms is a powerful result in itself ("absence of evidence") and serves as a necessary wake-up call for the field.

The manuscript is generally well-written, the methodology is robust (following PRISMA guidelines), and the conclusions are tempered and supported by the data. However, there are a few concerns regarding the currency of the search and the depth of the discussion that should be addressed prior to publication.

Major Comments:

1. The search strategy was updated to May 2024, yet the manuscript was submitted in July 2025. This represents a gap of more than 12 months. In rapidly evolving fields, or even for systematic reviews intended to set a benchmark, a gap of over a year can be considered a limitation.

- Recommendation:

Ideally, the authors should perform a quick update search (e.g., from May 2024 to present) to ensure no major recent studies have been missed. If this is not feasible, please provide a

strong justification in the Methods or Limitations section regarding why the findings are likely to remain unchanged despite this lag.

2. Definition and Categorization of "Harms": While the authors categorize harms (e.g., false confidence, cynicism, stress), it would be beneficial to clarify whether these categories were pre-defined based on a theoretical framework or if they emerged inductively during the data extraction process.

- Recommendation:

Please clarify the origin of these harm categories in the Methods section. If they were pre-defined, citing the relevant framework would strengthen the theoretical basis of the review.

3. Details on the Few Studies that Did Assess Harms: Although only a small fraction (10 studies) assessed harms, the readers would benefit from a slightly more detailed narrative description of how these specific studies attempted to measure them.

- Recommendation:

In the Results or Discussion, could the authors briefly expand on the measurement tools or methods used in those few positive instances? (e.g., Did they use validated scales for anxiety? Or open-ended qualitative questions?). This would add practical value for future researchers looking for guidance on how to start measuring these outcomes.

Minor Comments:

1. Abstract: The abstract is clear and concise. However, please ensure that the "Conclusion" in the abstract explicitly matches the nuanced conclusion in the main text regarding the distinction between "evidence of no harm" and "no evidence of harm."

2. Implications for Practice: The discussion on implications is strong. It might be strengthened by suggesting specific examples of potential adverse effects that researchers should prioritize in future logic models for critical thinking interventions.

3. Tables/Figures: Figure 1 (PRISMA flow diagram) is clear. Please ensure high-resolution versions are uploaded for the final production.

Conclusion: This is a high-quality methodological review that deserves publication after addressing the currency of the data and providing minor clarifications. I look forward to seeing the revised manuscript.

VERSION 1 - AUTHOR RESPONSE

REVIEWER 1 COMMENTS

Comment: “In the abstract, the authors say that this study ‘was an expansion of an earlier systematic review’ and then say it is the ‘first systematic review focusing on adverse effects of any category of educational interventions.’ This is confusing. Is this study the first review of its

kind or does it build upon a previous review? In general (in the abstract as well as in the introduction and discussion), the novelty of this study above and beyond what has been done previously should be more clearly highlighted."

Response: We agree with the comment and have revised the abstract and main text accordingly. What makes the study novel is that – to our knowledge – it is the first systematic review to assess the extent to which studies of any group of educational interventions assess potential adverse effects. The earlier review, which ours build upon, did not address potential adverse effects of the interventions.

Comment: "The introduction includes an example of a health intervention an educational intervention for reading with adverse effects but an example of an educational intervention for a health choice (and an adverse effect of that intervention) would be more useful."

Response: We have replaced the example about use of grades in school with an example about a secondary school intervention to think critically about health choices, from a recent article published in BMJ Open.

Comment: "I found Table 1 to be quite confusing. Are 1.1, 2.1, and 3.1 supposed to relate to each other since they're in the same row? Changing the orientation so that the row headers are column headers might help."

Response: We agree that the formatting of the table was confusing. No, concepts 1.1, 2.1, and 3.1 were only in the same row because they are the first concept in their respective groups. We have revised the table so the vertical lines between concept groups are visible, rather than the horizontal lines between concepts in the same group. Also, we placed all concepts in the same group in the same cell.

Comment: "The authors should address how the non-inclusion of unpublished studies might affect the results. It could be that those papers do assess and report adverse effects but that makes the paper/intervention seem weaker so it hasn't been published."

Response: This is an interesting point. We address it in the revised discussion section. We consider it unlikely that eligible studies would be unpublished or non-indexed due to a finding that the intervention was harmful. Rather, we consider it likely that any eligible studies reported in the grey literature did not assess any potential adverse effect, given the results of the review suggest this is overwhelmingly the case. Note that we did include an unpublished poster reporting a qualitative study, in which the authors concluded, "None of the results indicated that the intervention caused side effects" (Steckelberg 2008).

Comment: "The authors should clearly define what an adverse effect is in this context. On p. 6, they say 'we extracted any mention of potential adverse effects' but it is not clear how they decided whether something was an adverse effect. In other words, what instructions were coders given on how to identify a mention of an adverse effect? Did the phrase adverse effect specifically need to be mentioned or could an adverse effect be inferred from context clues?"

Response: We agree that we have been unclear in this area. We had already defined adverse effect (an increase in adverse outcomes or decrease in beneficial outcomes) in the introduction and repeated the definition once in the methods section. In the methods section, we had stated that we extracted data based on the definition, not the use of specific terms. This was imprecise; we extracted data based on the definition *and use of relevant terminology*, but not

the specific use of certain terms. We have added relevant clarification several places in the section on data extraction.

Comment: "It would be useful to add a column to Table 4 showing which of the 'Key concepts for informed health choices' that study addressed."

Response: Like in the earlier review, we only assessed whether the intervention was intended to improve understanding of any of the key concepts and did not create an overview of each concept addressed by each intervention. We agree that such an overview would be useful context to an updated analysis of intended effects, but do not believe that it would be particularly useful in the context of assessing the extent to which the studies assessed potential harms.

Comment: "In Table 5, are the results presented the results of the adverse effect? Meaning, there was no differences between intervention groups on their experience of the potential adverse effect? This should be made more clear."

Response: Yes, the results are from assessing the adverse effect. There was a difference between the groups in terms of the relevant outcome, but – based on the confidence interval – neither a difference showing the intervention had the adverse effect nor a difference showing the intervention did not have such an effect. We agree that this was unclear in our original manuscript and have added explanation to the main text.

Comment: "The authors might consider bolding the text in Table 6 that relates to adverse effects as some of the quotes are long and some of the text is introductory rather than substantive."

Response: Excellent idea. We have bolded relevant text in the table, as suggested.

Comment: "At the top of p. 13, the authors report that the number of studies as well as the percentage (1/35, 3%) that meet the criteria for that analysis. For consistency, the authors might consider adding a percentage to the result in the middle of p. 13 and at the top of p. 16."

Response: We have added the percentages where suggested.

Comment: "Based on Table 7, it seems that many protocols mention the possibility of adverse effects but then those effects (even the absence of them) are not reported in the published studies. The authors should discuss this disconnect between the protocol and the published study. Is it possible that adverse effects were not mentioned because there were no adverse effects in that study? Do the authors recommend that all studies include a statement about adverse effects, even just to acknowledge that there were none?"

Response: This is an important comment. The referenced part of the text in the original manuscript was unclear and imprecise. One problem that led to Table 7 being unclear is that some of the associated qualitative studies were in fact multi/mixed-methods, but we treated these as qualitative studies, *except in Table 7*. In the revised manuscript, we have explained explicitly how we dealt with associated, separately-reported qualitative studies that were in fact multi/mixed-methods and revised Table 7 to accurately reflect the analysis. Another problem that led to Table 7 being unclear was that the text above the table was imprecise: if the protocol mentioned potential adverse effects, the report indeed mentioned potential adverse effects. However, only the one protocol mentioned potential adverse effects in the context of plans for assessing a specific potential harm, in a comparison between interventions. We have revised the text above Table 7 accordingly. Moreover, two investigators have independently reviewed the

other contexts in which protocols mentioned potential adverse effects and incorporated this information in the main text and table. We recommend that all relevant study reports address potential adverse effects. We have added this important point to the discussion of implications.

Comment: “The ‘Other evidence’ section feels out of place. Most of the evidence in this section does not relate to health choices/interventions. If the authors wish to keep this section, they need to make it more clear how this relates to the findings they present earlier.

Response: In the revised manuscript, we have removed the last two paragraphs in the “Other evidence” section.

Comment: “In the ‘Strengths’ section, the authors conclude ‘the results clearly suggest researchers generally have not considered or assessed potential adverse effects…’ However, Table 7 suggests that most researchers have considered potential adverse effects but have not reported them in their published study.”

Response: Another important comment. The quoted statement was imprecise. We have revised the sentence in the “Strenghts” section and elsewhere: the results clearly suggest that researchers generally have not assessed potential adverse effects of relevant interventions (although they may have considered such effects).

Comment: “The table(s) presented in the Supplementary Files starting on page 52 of 131 of the PDF are very difficult to piece together across multiple pages.”

Response: We are sorry that the tables were difficult to piece together. We submitted the relevant supplementary files as Microsoft Excel spreadsheets. For the revised submission, we uploaded the spreadsheets to Zenodo and, where relevant in the revised manuscript, added references to the upload.

REVIEWER 2 COMMENTS

Comment: “The search strategy was updated to May 2024, yet the manuscript was submitted in July 2025. This represents a gap of more than 12 months. In rapidly evolving fields, or even for systematic reviews intended to set a benchmark, a gap of over a year can be considered a limitation.”

Recommendation: “Ideally, the authors should perform a quick update search (e.g., from May 2024 to present) to ensure no major recent studies have been missed. If this is not feasible, please provide a strong justification in the Methods or Limitations section regarding why the findings are likely to remain unchanged despite this lag.”

Response: We appreciate that it might be unusual with two updates to the search reported in the same article. It is correct that we submitted the article in July 2025. However, the last update to the search was up to March 2025, not May 2024. We have not mentioned May 2024 anywhere in the manuscript. Perhaps the reviewer is referring to the search in Web of Science up to May 2023, as part of the first update to the search? Note that we first submitted the article to BMJ Open in December 2024, but it was declined due to the search being over 18 months old. We then updated the search and resubmitted.

Comment: “Definition and Categorization of ‘Harms’: While the authors categorize harms (e.g., false confidence, cynicism, stress), it would be beneficial to clarify whether these categories were pre-defined based on a theoretical framework or if they emerged inductively during the data extraction process.”

Recommendation: “Please clarify the origin of these harm categories in the Methods section. If they were pre-defined, citing the relevant framework would strengthen the theoretical basis of the review.”

Response: Perhaps the reviewer is referring to a framework described in a separate paper that we reference at the end of the “Implications” section? We did not use that framework or any other frameworks in the review. In the “Data extraction” section, we have clarified an adverse effect could be an increase in any adverse outcome, or decrease in any beneficial outcome, and that we took authors’ descriptions of effects as adverse at face value.

Comment: “Details on the Few Studies that Did Assess Harms: Although only a small fraction (10 studies) assessed harms, the readers would benefit from a slightly more detailed narrative description of how these specific studies attempted to measure them.”

Recommendation: “In the Results or Discussion, could the authors briefly expand on the measurement tools or methods used in those few positive instances? (e.g., Did they use validated scales for anxiety? Or open-ended qualitative questions?). This would add practical value for future researchers looking for guidance on how to start measuring these outcomes.”

Response: This comment relates to comments by Reviewer 1 about Tables 5 and 7, revealing a lack of clarity in the original manuscript. We only found one study in which researchers assessed any potential adverse effect of a relevant intervention. We have added a description of the assessment, including the outcome measure. As explained in a response to Reviewer 1, we have revised the text preceding Table 7 and the content of Table 7, to make it clear that only one of the protocols included plans to assess a specific potential harm, using a specific outcome measure.

Comment: “The abstract is clear and concise. However, please ensure that the ‘Conclusion’ in the abstract explicitly matches the nuanced conclusion in the main text regarding the distinction between ‘evidence of no harm’ and ‘no evidence of harm.’

Response: We have adjusted the conclusion in the abstract: we showed that researchers generally have not assessed potential adverse effects of relevant interventions (although they may have considered them).

Comment: “The discussion on implications is strong. It might be strengthened by suggesting specific examples of potential adverse effects that researchers should prioritize in future logic models for critical thinking interventions.”

Response: We agree. We have added the categories of potential adverse effects from the aforementioned framework, with a reference to an article describing the revision of the framework. The article was published in BMJ Open after submitting the original manuscript for the review.

Comment: “Tables/Figures: Figure 1 (PRISMA flow diagram) is clear. Please ensure high-resolution versions are uploaded for the final production.”

Response: We will ensure that the versions of the figures uploaded for the final production are high enough resolution, based on the journal’s guidelines.

VERSION 2 - REVIEW

**Reviewer**	**1**
**Name**	**Vogt, Randi**
**Affiliation**	**Geisinger, Bioethics and Decisions Sciences**
**Date**	**12-Jan-2026**
**COI**	

The authors did a nice job on this revision. I have no further comments.

**Reviewer**	**2**
**Name**	**Sornlorm, Kittipong**
**Affiliation**	**Khon Kaen University, Faculty of Public Health, Khon Kaen University**
**Date**	**06-Jan-2026**
**COI**	

1. Critical Conflict of Interest Issue You acknowledge that "many of the reports...had one or more of the same authors, including investigators in this review" this is severely understated. The Informed Health Choices studies (Nsangi, Semakula, Ssenyonga, Chesire, Mugisha) constitute a substantial portion of included studies and share authorship with this review team. You are essentially concluding your own research program appropriately assessed harms while others failed. This requires explicit COI declaration and independent extraction of your own studies.

2. Circular Methodology You searched without harm-related terms, then concluded harm assessment is rare. This is methodologically flawed. Conduct a sensitivity search with "adverse," "harm," "unintended effects" to verify completeness.

3. Misleading Headline Finding The 3% (1/35) quantitative assessment rate obscures that:

- 67% of qualitative studies explored harms

- 73% of protocols mentioned harms

Researchers clearly consider harms — they simply don't measure them quantitatively. Your conclusion overstates the problem.

4. Vague Operationalization What distinguishes an "adverse effect" from a barrier, limitation, or negative feedback? Your extraction criteria remain unclear despite revision. The conflation of quantitative assessment, qualitative exploration, safety monitoring, and protocol mentions undermines interpretability.

5. Missing Benchmarks Is 3% uniquely low? Without comparison to harm reporting rates in behavioral interventions, drug trials, or other educational research, readers cannot evaluate significance.

6. Weak Implications "Pay more attention to harms" is insufficient guidance. Which harms warrant priority? What instruments exist? What designs are needed? Your own framework is mentioned but not operationalized for readers.

VERSION 2 - AUTHOR RESPONSE

REVIEWER 1

Comment: "The authors did a nice job on this revision. I have no further comments."

Response: Thank you. Your critical comments on the original manuscript were very clear and helpful.

REVIEWER 2

Comment: "1. Critical Conflict of Interest Issue You acknowledge that 'many of the reports...had one or more of the same authors, including investigators in this review' this is severely understated. The Informed Health Choices studies (Nsangi, Semakula, Ssenyonga, Chesire, Mugisha) constitute a substantial portion of included studies and share authorship with this review team. You are essentially concluding your own research program appropriately assessed harms while others failed. This requires explicit COI declaration and independent extraction of your own studies."

Response: We agree that the overlapping authorship is an important point and that we should have mentioned it in the section on competing interests as well. We have revised the section accordingly. However, we cannot see that the extent of the overlap as worded in the manuscript ("many of the reports") constitutes an understatement compared to the wording in the comment ("a substantial portion of the included studies").

We agree that it would be problematic to conclude that studies in which we have been involved have assessed harms appropriately, while others have failed. However, we have not made such a conclusion either explicitly or implicitly. Rather, our conclusion is that researchers generally have not assessed potential harms of relevant interventions. The conclusion applies to our own research. In the new manuscript (second revision), in response to the comment, we have made this point explicit in the discussion of principal findings.

Two reviewers independently extracted all data for the review, and we have made all the data underlying the review available in a public repository. Most of the authors of the review, including authors who contributed to data extraction, were not authors of any of the included studies. We believe further data extraction is unnecessary and would be a poor use of time and resources.

Comment: "2. Circular Methodology You searched without harm-related terms, then concluded harm assessment is rare. This is methodologically flawed. Conduct a sensitivity search with 'adverse,' 'harm,' 'unintended effects' to verify completeness."

Response: In the new discussion section, we address that we did not use search terms specifically for harms, in addition to providing our rationale in the methods. It is unlikely that there are studies in which researchers have only assessed potential adverse effects of relevant interventions, without assessing any intended effect. If there were studies limited to assessing potential harms of relevant interventions, we likely would have found them with the search terms that we did use or discovered references to those studies during data extraction. Hence, we believe a sensitivity search too is unnecessary and would be a poor use of time and resources.

Comment: “3. Misleading Headline Finding The 3% (1/35) quantitative assessment rate obscures that:
- 67% of qualitative studies explored harms
- 73% of protocols mentioned harms

Researchers clearly consider harms — they simply don't measure them quantitatively. Your conclusion overstates the problem.”

Response: This comment reveals a lack of clarity in the manuscript, which we have revised accordingly. We disagree that the results show researchers generally consider harms of relevant interventions. What the results do suggest is that when researchers have conducted mixed/multi-methods or associated qualitative studies, they typically describe exploring experiences of potential adverse effects. However, they typically have <u>not</u> conducted a mixed/multi-methods study or associated qualitative study. Similarly, when researchers have published a protocol for the quantitative study, the protocol typically mentions potential adverse effects, but typically the researchers have <u>not</u> published a protocol.

Comment: “4. Vague Operationalization What distinguishes an ‘adverse effect’ from a barrier, limitation, or negative feedback? Your extraction criteria remain unclear despite revision. The conflation of quantitative assessment, qualitative exploration, safety monitoring, and protocol mentions undermines interpretability.”

Response: We agree that the manuscript was still missing clarity in this area and have revised the methods and results sections accordingly. In both sections, we have distinguished between “primary” analysis (reports of quantitative studies) and “secondary” analyses (reports of qualitative studies, and protocols for quantitative and qualitative studies). In the methods section, we have defined not just what we mean by adverse effect, but what we mean by assessing an adverse effect, and we have added detail about how we extracted data from qualitative studies. In the results, we have addressed the mix of findings about potential harms and findings about intervention limitations and barriers in the study by Berget et al.

Comment: “5. Missing Benchmarks Is 3% uniquely low? Without comparison to harm reporting rates in behavioral interventions, drug trials, or other educational research, readers cannot evaluate significance.”

Response: We disagree with the comment. One of 35 reports (3%) is on its face a low number, regardless of rates for other types of interventions. Furthermore, it does not make sense to compare our results to results for reports of drug trials. And, as noted in the discussion, to our knowledge, our review is the first of its kind in educational research.

Comment: “6. Weak Implications ‘Pay more attention to harms’ is insufficient guidance. Which harms warrant priority? What instruments exist? What designs are needed? Your own framework is mentioned but not operationalized for readers.”

Response: In the section on implications, we detail what we mean by paying more attention to potential harms. The questions in the comment are beyond the scope of the review. However, we have added information related to use of the framework.
